# High-Throughput Sequencing Study of the Mycobiota of Barley Grain Collected in Northern and Central Italy and Accumulation of Fungal Secondary Metabolites

**DOI:** 10.3390/toxins18090363

**Published:** 2026-08-25

**Authors:** Lorenzo Covarelli, Giovanni Beccari, Emilio Balducci, Ida Karlsson, Paula Persson, Hanna Friberg, Michael Sulyok, Antonio Prodi, Massimo Montanari, Francesco Tini

**Affiliations:** 1 Department of Agricultural, Food and Environmental Sciences, University of Perugia, 06121 Perugia, Italy; lorenzo.covarelli@unipg.it (L.C.); emilio.balducci@unipg.it (E.B.); francesco.tini@unipg.it (F.T.); 2 Department of Crop Production Ecology, Swedish University of Agricultural Sciences, 75007 Uppsala, Sweden; ida.karlsson@scilifelab.uu.se (I.K.); 3 Department of Immunology, Genetics and Pathology, Uppsala University, 75185 Uppsala, Sweden; 4 Department of Forest Mycology and Plant Pathology, Swedish University of Agricultural Sciences, 75007 Uppsala, Sweden; hanna.friberg@slu.se; 5 Department of Agricultural Sciences, Institute of Bioanalytics and Agro-Metabolomics, BOKU University, 3430 Tulln, Austria; michael.sulyok@boku.ac.at; 6 Department of Agricultural and Food Sciences, University of Bologna, 40127 Bologna, Italy; antonio.prodi@unibo.it; 7 Council for Agricultural Research and Economics (CREA), Research Centre for Cereal and Industrial Crops, 40128 Bologna, Italy; massimo.montanari@crea.gov.it

**Keywords:** barley, *Ascomycota*, *Basidiomycota*, *Fusarium*, *Alternaria*, mycotoxins

## Abstract

Barley grain provides an ecological niche in which fungal microorganisms can develop. The presence of mycotoxigenic genera can result in the accumulation of fungal secondary metabolites. The present study aims to survey barley grain samples collected from Northern and Central Italy by assessing: (1) the presence of fungal microorganisms using high-throughput sequencing of the *internal transcribed spacer* region; (2) the presence and quantification, by real-time quantitative PCR (RT-qPCR), of the main *Fusarium* species associated with *Fusarium* head blight; and (3) the presence and quantification of secondary metabolites produced by *Fusarium* and *Alternaria* using liquid chromatography–tandem mass spectrometry. Both potentially pathogenic genera (mainly within the *Ascomycota* division) and non-pathogenic genera (within both *Ascomycota* and *Basidiomycota* divisions) were detected. *Alternaria* and *Cryptococcus* were the dominant genera within *Ascomycota* and *Basidiomycota* divisions, respectively, in both macro-areas. Some genera, such as *Aureobasidium* and *Cryptococcus*, showed interesting negative correlations with several pathogenic genera. Two important mycotoxigenic genera (*Alternaria* and *Fusarium*) were detected, with RT-qPCR that revealed the predominance of *Fusarium graminearum* in the Northern macro-area. *Fusarium* secondary metabolites exhibited generally higher accumulation in the North, whereas *Alternaria* secondary metabolites were more evenly distributed across Italy, reflecting the abundance of the two genera. The co-occurrence of *Fusarium* and *Alternaria* secondary metabolites within single barley grain samples was also observed.

## 1. Introduction

Barley (*Hordeum vulgare* L.) is one of the most widely cultivated cereals worldwide, with 46 million hectares and 146 million tons produced in 2023 [1]. In Italy, barley covers about 290,000 hectares and yields 1.2 million tons in 2023 [1]. Although mainly used for animal feed, it is also the key ingredient in the beer industry [2], and to a lesser extent for human consumption [3].

Barley spikes and grain provide an ecological niche that supports the development of various microorganisms, particularly fungi belonging to the *Ascomycota* and *Basidiomycota*, including both endophytic and non-endophytic taxa, as well as pathogenic and non-pathogenic taxa [4,5,6,7,8,9,10,11,12,13,14,15,16,17,18,19].

Commonly, the quality and quantity of barley grain are compromised by a wide range of *Ascomycota* seed-borne fungi, including species within the genera *Fusarium*, *Alternaria*, *Microdochium* and *Pyrenophora* [20,21,22,23,24,25]. Various *Fusarium* species responsible for *Fusarium* Head Blight (FHB) affect seeds, causing them to shrivel and bleach [26]. *Microdochium* can also cause symptoms on grain similar to those induced by *Fusarium* species [27]. *Alternaria* species act as major causal agents of sooty mold and black point, diseases to which other fungal genera, such as *Cladosporium*, may also contribute [28,29]. Although *Pyrenophora* does not typically cause visible symptoms on seeds, it is seed-transmitted to young plants, impacting them from the earliest developmental stages [20,21,30].

Among the previously mentioned genera, *Alternaria* and *Fusarium* are typically the dominant members of the barley grain mycobiota in various cultivation areas, including Italy [22,23,24]. Some species belonging to these two genera can produce mycotoxins, secondary metabolites that pose significant health risks to humans and animals. Due to the very harmful effects of some molecules, such as deoxynivalenol (DON), T-2 and HT-2 toxins and zearalenone (ZEN), all produced by *Fusarium*, their legal limits in grain and resulting processed food are regulated by the European Union [31,32,33]. In addition, some *Alternaria* mycotoxins, including alternariol (AOH), alternariol monomethyl ether (AME), and tenuazonic acid (TeA), have been mentioned in a European recommendation [34].

Recently, the co-occurrence of *Fusarium* and *Alternaria* in barley grain has been reported, together with the simultaneous presence of mycotoxins produced by these genera [32]. Several studies have highlighted that mixtures of mycotoxins may exert additive, synergistic, or interactive effects, suggesting that combined exposure could represent a relevant concern for human and animal health. The potential risk of chronic exposure to multiple mycotoxins from co-contamination in food may lead to a greater toxicity than exposure to a single mycotoxin [33].

Until a decade ago, despite the wide array of microorganisms present in cereal grain, only a limited number of genera could typically be identified and quantified. This was largely caused by the limitations of culture-dependent methods, which are generally performed using only a few media or techniques for isolation under a single condition, while a broader range of conditions, media, and techniques would be required to capture a larger diversity of microorganisms [12,34,35]. However, for decades, cultivation-based methods have provided an important insight into geographical distribution, as well as biodiversity [6]. It should also be noted that, although diagnostic techniques based on microorganism isolation may provide only a limited view of the fungal species associated with a given matrix, these approaches allow the recovery of isolates that can subsequently be further characterized from multiple perspectives. However, such techniques do not enable the detection of fungal species that are non-culturable or difficult to culture. To address these limitations, real-time quantitative PCR (RT-qPCR) assays performed directly on grain material enable the identification of fungal species that grow, do not grow, or grow only poorly *in vitro*. Nevertheless, these methods are constrained by the choice of species-specific primers, which restricts the analysis to a predefined set of target fungi, often selected based on prior isolation work or expected species [36,37].

In the last decade, together with culture methods and more traditional molecular biology techniques (RT-qPCR), other techniques such as high-throughput sequencing (HTS) technologies have been frequently used [38,39,40]. These techniques are becoming an indispensable tool in fungal community ecology analysis, and sequencing of the *internal transcribed spacer* (*ITS*) region of ribosomal fungal DNA [41] allows identification down to the genus level [42,43,44]. Compared with the previously described techniques, HTS enables the detection of different microorganisms within single samples, including unculturable or low abundance fungi [45,46]. Combining HTS with targeted RT-qPCR assays can provide a more comprehensive view of the mycobiota in a plant matrix [38].

As previously mentioned, barley is one of the most widely cultivated cereals in Italy [1]. It is well known that Italy can be subdivided into three agroclimatic macro-areas characterized by distinct climatic conditions, ranging from the cool and wet climates of Northern Italy to the warmer and drier climates of Southern Italy [47]. These contrasting conditions may increase or reduce the occurrence of fungal microorganisms depending on how favorable they are for fungal development, as previously observed in cereal crops [48]. Despite several studies describing barley-associated fungi through culture-dependent or single-method approaches, an integrated characterization of the barley grain mycobiota combining HTS, RT-qPCR and liquid chromatography tandem mass spectrometry (LC-MS/MS) remains relatively uncommon. To the best of our knowledge, only limited information is available on studies that simultaneously assessed fungal community structure, key *Fusarium* species and secondary metabolites across Italian macro-areas. Given the agronomic and industrial relevance of barley cultivation in Italy, as well as the climatic variability across the country and among years, further investigations are needed to clarify and update our understanding of how fungal communities relate to mycotoxin contamination. The integrated approach adopted in this study provides novel and complementary insights into the Italian barley mycobiota and its associated secondary metabolites.

Considering what has been outlined so far, this study aims to survey 54 barley grain samples collected in 2019 from different cultivation areas in Northern and Central Italy, by assessing: (1) the fungal microorganisms associated with the grain samples using HTS of the *ITS* region of fungal ribosomal DNA, allowing identification at the genus level; (2) the presence and quantification, by RT-qPCR, of the eight main *Fusarium* species typically associated with FHB; (3) the presence and quantification of secondary metabolites produced by *Fusarium* and *Alternaria* species using LC-MS/MS.

## 2. Results

### 2.1. Fungal Microorganisms Associated with the Barley Grain Samples Determined by High-Throughput Sequencing of the ITS Region of Ribosomal Fungal DNA

**Figure 1:**
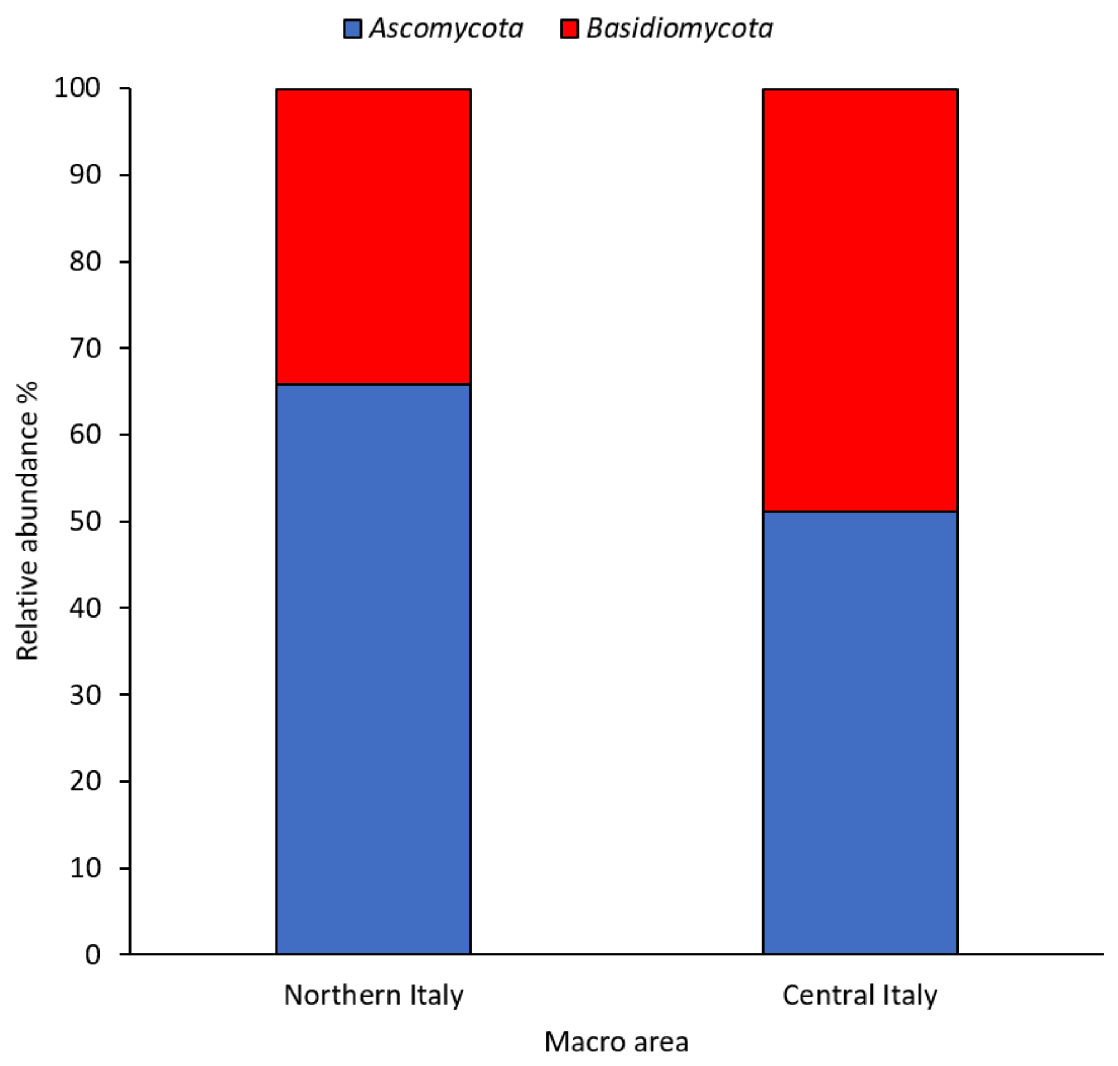
Relative abundance (%) of *Ascomycota* and *Basidiomycota* divisions in barley grain samples collected in the macro-areas of Northern and Central Italy in 2019, as determined by high-throughput sequencing of the *ITS* region of fungal ribosomal DNA.

Barley grain samples were mainly colonized by *Ascomycota*, which were more abundant than *Basidiomycota* in both macro-areas. However, this difference was significant (*p* < 0.05) only in Northern Italy (Figure 1, Table S1).

**Figure 2:**
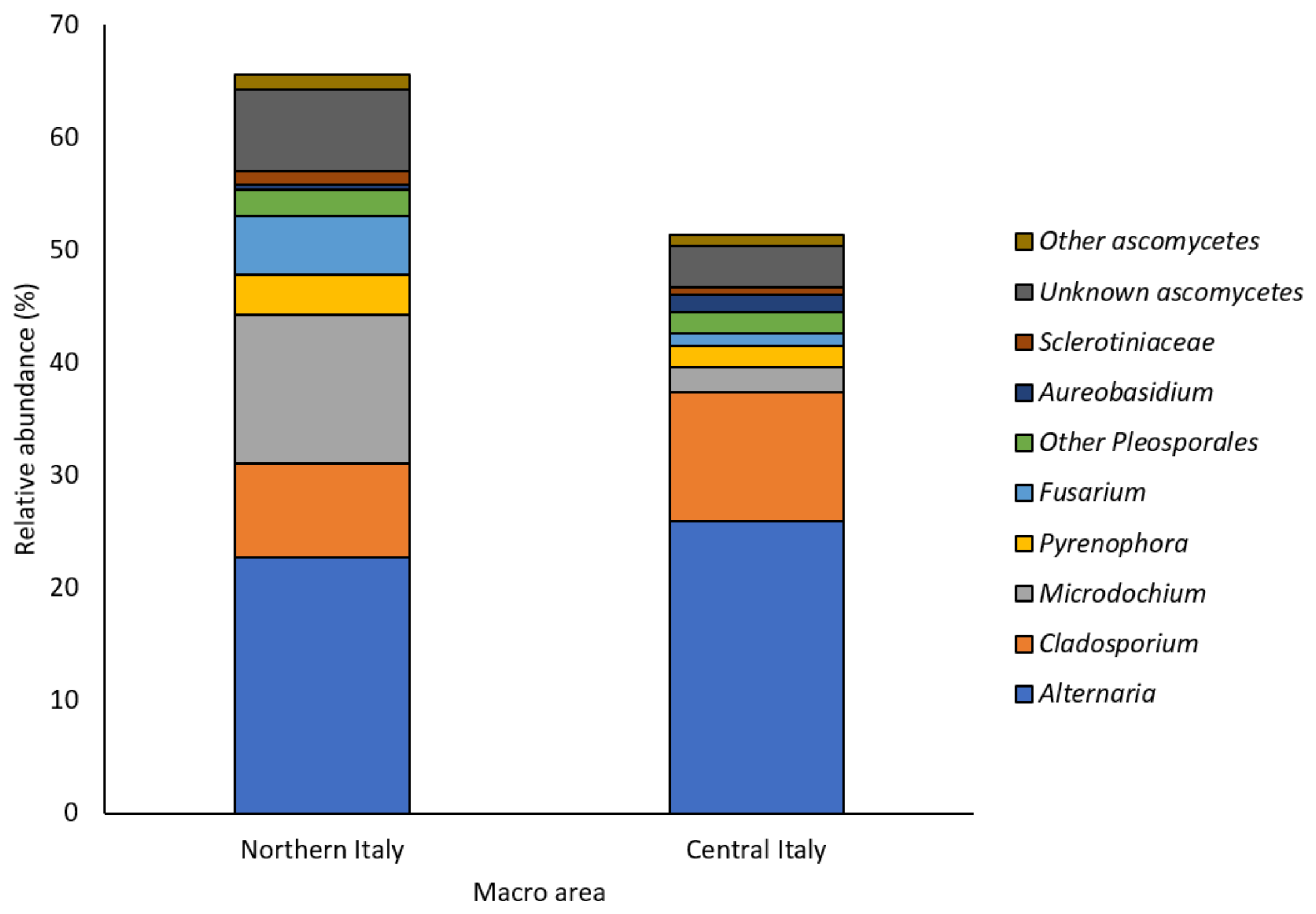
Relative abundance (%) of fungal microorganisms belonging to the *Ascomycota* division in barley grain samples collected in the macro-areas of Northern and Central Italy in 2019, as determined by high-throughput sequencing of the *ITS* region of fungal ribosomal DNA.

Within *Ascomycota*, a total of six genera were identified: *Alternaria*, *Aureobasidium*, *Davidiella*, *Fusarium*, *Monographella*, and *Pyrenophora*. For statistical analysis, operational taxonomic units (OTUs) were grouped at the genus level when possible. According to the “one fungus = one name” principle [49,50], teleomorphic names were replaced by their corresponding anamorphic genera; therefore, *Davidiella* and *Monographella* were reported as *Cladosporium* and *Microdochium*, respectively (Figure 2). For some microorganisms, the technique used did not allow identification at the genus level, but in certain cases, it was possible only at the order level (*Pleosporales*) or at the family level (*Sclerotiniaceae*). It should be noted that, since *Alternaria* and *Pyrenophora* are genera within the order *Pleosporales*, and these genera could be identified using the applied technique, it is considered to fall within the genus category. Therefore, the order *Pleosporales* should include all microorganisms excluding *Alternaria* and *Pyrenophora*, and for this reason, named “other *Pleosporales*”. In addition, those genera with a very low relative abundance (<0.038) were grouped into the category “other ascomycetes”, whereas those microorganisms for which it was not possible to identify the genus, order, or family were classified as “unknown ascomycetes” (Figure 2, Table S2).

In samples from Northern Italy, *Alternaria* was the predominant genus detected (*p* < 0.05). *Alternaria* was also the most prevalent *Ascomycota* genus in samples from Central Italy (*p* < 0.05) (Figure 2, Table S2).

Focusing on the most noteworthy potentially pathogenic genera of barley, *Alternaria* exhibited similar relative abundance in samples from both macro-areas (*p* > 0.05), as well as *Pyrenophora* (*p* > 0.05). In contrast, *Microdochium* and *Fusarium* were both more prevalent in Northern Italy than in Central Italy (*p* < 0.05).

**Figure 3:**
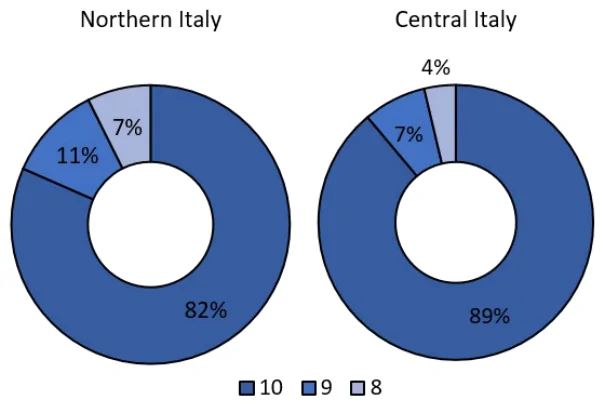
Percentage of barley samples collected in the macro-areas of Northern and Central Italy in 2019 showing co-occurrence (in a single sample) of 10, 9, or 8 categories of fungal microorganisms belonging to the *Ascomycota* division, as determined by high-throughput sequencing of the *ITS* region of fungal ribosomal DNA.

The distribution of *Ascomycota* categories in individual samples is shown in Figures S1 and S2. Most barley grains contained a broad set of *Ascomycota* genera, with all ten categories co-occurring in the majority of samples from both macro-areas. Samples with nine or eight categories were also frequent, confirming the high complexity of the barley mycobiota (Figure 3).

**Figure 4:**
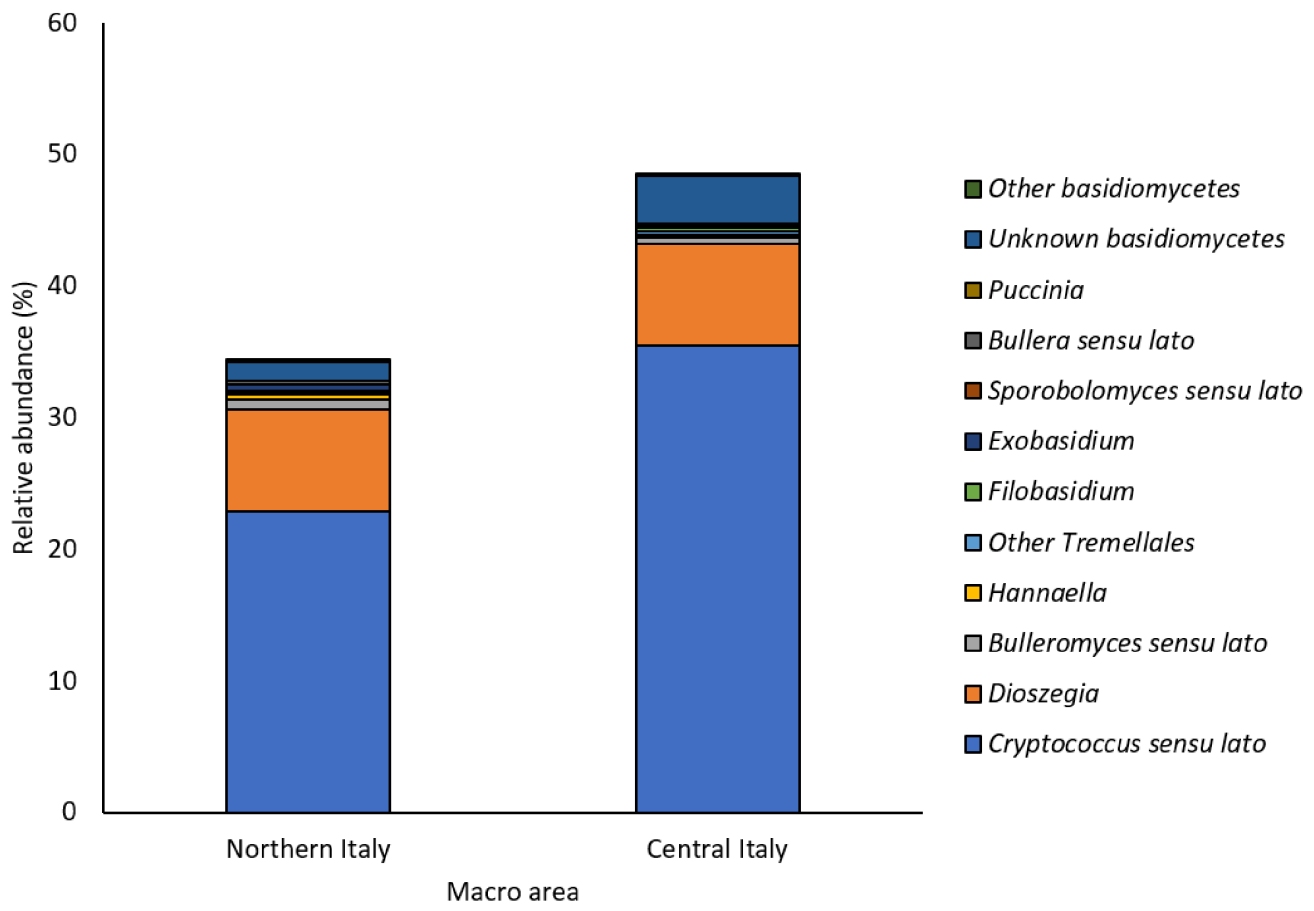
Relative abundance (%) of fungal microorganisms belonging to the *Basidiomycota* division in barley grain samples collected in the macro-areas of Northern and Central Italy in 2019, as determined by high-throughput sequencing of the *ITS* region of fungal ribosomal DNA.

Focusing on the *Basidiomycota* division, a total of nine genera were identified: *Bullera*, *Bulleromyces*, *Cryptococcus*, *Dioszegia*, *Exobasidium*, *Filobasidium*, *Hannaella*, *Puccinia*, and *Sporobolomyces*. Also, in this case, taxonomic assignments were performed according to the “one fungus = one name” principle [49,50]. When *ITS* sequences could not be reliably resolved at the updated genus level (*Bullera*, *Bulleromyces*, *Cryptococcus*, *Sporobolomyces*), taxa were reported sensu lato (s.l.) to ensure consistency with current fungal taxonomy. Although the genera *Bullera* and *Bulleromyces* are currently considered phylogenetically congruent with *Hannaella* [51], sequences assigned to *Bullera* and *Bulleromyces* were retained as *Bullera* s.l. and *Bulleromyces* s.l., as *ITS* data did not allow a reliable reassignment to *Hannaella* sensu stricto (s.s.). For some microorganisms, the technique used did not allow identification at the genus level, but in certain cases, it was possible only at the order level (*Tremellales*). It should be noted that, since *Bullera* s.l., *Bulleromyces* s.l., *Cryptococcus* s.l., *Dioszegia*, *Filobasidium*, *Hannaella*, and *Sporobolomyces* s.l. are genera within the order *Tremellales*, and these genera were correctly identified using the applied technique, they are considered to fall within the genus category. Therefore, the order *Tremellales* should include all microorganisms excluding *Bullera* s.l., *Bulleromyces* s.l., *Cryptococcus* s.l., *Dioszegia*, *Filobasidium*, *Hannaella*, *Sporobolomyces* s.l. and, for this reason, named “other *Tremellales*”. In addition, those genera with a very low relative abundance (<0.76%) were grouped into the category “other basidiomycetes”, whereas those microorganisms for which it was not possible to identify the genus, order, or family were classified as “unknown basidiomycetes” (Figure 4, Table S3).

*Cryptococcus* s.l. was the basidiomycete with the highest relative abundance (*p* < 0.05) in both macro-areas, and it was significantly more abundant in samples from Central Italy (Figure 4, Table S3).

*Dioszegia* was the second most abundant basidiomycete in both macro-areas, showing levels significantly lower than *Cryptococcus* s.l. but higher than all other genera (*p* < 0.05), with no differences between Northern and Central Italy.

The remaining basidiomycetes occurred at lower and comparable abundances, although some genera displayed regional variation: *Bullera* s.l. and *Exobasidium* were more frequent in Northern Italy, whereas *Filobasidium*, *Puccinia*, other *Tremellales* and unknown basidiomycetes were more abundant in Central Italy.

**Figure 5:**
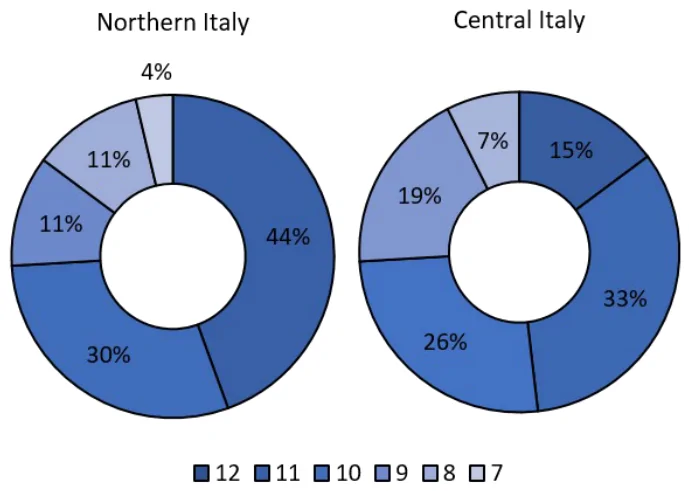
Percentage of barley samples collected in the macro-areas of Northern and Central Italy in 2019 showing co-presence (in a single sample) of 12, 11, 10, 9, 8, or 7 categories of fungal microorganisms belonging to the *Basidiomycota* division, as determined by high-throughput sequencing of the *ITS* region of fungal ribosomal DNA.

The relative abundance of microorganisms belonging to the *Basidiomycota* division in each analyzed sample from Northern Italy and Central Italy is shown in Figures S3 and S4, respectively. A high number of basidiomycete categories frequently co-occurred within single barley grains, with 10–12 categories often present simultaneously (Figure 5).

### 2.2. Relationship Between Potentially Pathogenic Fungal Genera and Other Components of the Barley Mycobiota

Hereafter, the relationship between selected potentially pathogenic fungal genera affecting barley grain (*Alternaria*, *Cladosporium*, *Microdochium*, *Pyrenophora*, *Fusarium*) and other components of the barley mycobiota is presented. All correlations among fungal genera are shown in Table 1, while the corresponding *p*-values are shown in Table S4.

Focusing on *Alternaria* (involved in sooty mold and black point [29]), the strongest significant (*p* < 0.05) positive correlations were noticed with *Cladosporium*, other *Pleosporales*, and *Filobasidium*. On the other hand, the strongest significant (*p* < 0.05) negative correlations were observed with: *Fusarium*, *Microdochium*, *Bulleromyces* s.l., *Hannaella*, *Sporobolomyces* s.l. and unknown basidiomycetes.

*Cladosporium* (another genus involved in black point [29]) showed the strongest significant (*p* < 0.05) negative correlations with *Cryptococcus* s.l., *Hannaella*, *Bullera* s.l. and other basidiomycetes. Whereas the strongest significant (*p* < 0.05) positive correlations were detected with *Alternaria*, *Aureobasidium*, and *Filobasidium*.

*Fusarium* (a fungal genus that includes several FHB-causing species) showed the strongest significant (*p* < 0.05) negative correlation with *Alternaria*, *Aureobasidium*, *Cryptococcus* s.l., *Filobasidium* and *Puccinia*. Conversely, this genus showed the strongest significant (*p* < 0.05) positive correlation with *Microdochium*, other ascomycetes, *Exobasidium*, and *Bulleromyces* s.l.

Focusing on *Microdochium* (a fungal genus with species able to cause head blight symptoms [30]), this genus showed the strongest significant (*p* < 0.05) negative correlation with *Alternaria*, *Aureobasidium*, *Cryptococcus* s.l., other *Tremellales*, *Filobasidium*, *Sporobolomyces* s.l. and *Puccinia*, and unknown basidiomycetes. Conversely, this genus showed the strongest significant (*p* < 0.05) positive correlations with *Fusarium* and *Exobasidium*.

About *Pyrenophora* (a seed-transmitted fungal pathogen able to cause net blotch of barley [21]), this genus showed the strongest significant (*p* < 0.05) negative correlations with *Cryptococcus* s.l. Conversely, this genus showed the strongest significant (*p* < 0.05) positive correlations with unknown ascomycetes and other basidiomycetes.

### 2.3. DNA Accumulation of Eight Fusarium Species in Barley Grains

The R^2^ values and efficiency of the RT-qPCR reactions are summarized in Table S5.

**Figure 6:**
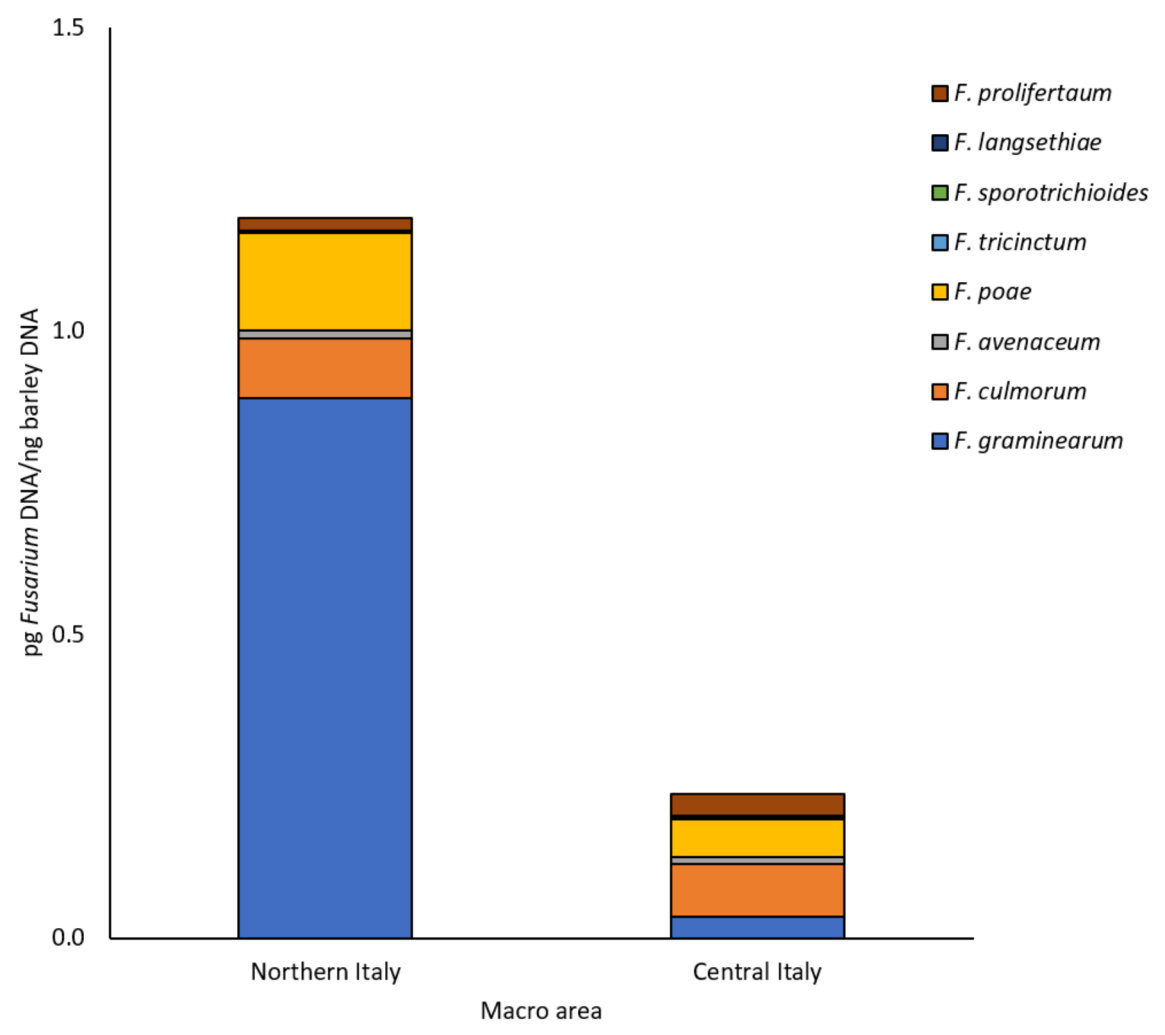
DNA amount (pg of each analyzed fungal species DNA/ng of barley) of eight *Fusarium* species in barley grain samples collected in the macro-areas of Northern and Central Italy in 2019 as determined by quantitative real-time polymerase chain reaction (RT-qPCR). Colors assigned to *F. sporotrichioides* and *F. tricinctum* are shown for completeness, although these species were not detected in any of the analyzed samples.

The fungal DNA of the eight *Fusarium* species accumulated in the barley grain samples is shown in Figure 6 and in Table S6.

Considering all eight *Fusarium* species, their overall DNA levels were higher in Northern Italy. In Northern samples, *Fusarium graminearum* was clearly the most abundant species (*p* < 0.05), while the other *Fusarium* species showed similar and lower DNA levels; *Fusariarium sporotrichioides* and *Fusarium tricinctum* were not detected. In Central Italy, no significant differences were observed among species. Only *F. graminearum* and *Fusarium poae* differed significantly (*p* < 0.05) between macro-areas, both being more abundant in Northern Italy.

**Figure 7:**
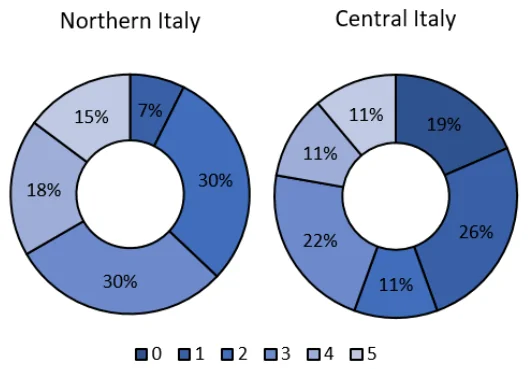
Percentage of barley samples collected in the macro-areas of Northern and Central Italy in 2019 showing co-presence (in a single sample) of 0, 1, 2, 3, 4 or 5 *Fusarium* species, as determined by quantitative real-time polymerase chain reaction (RT-qPCR).

*Fusarium* species DNA accumulation in each sample from Northern and Central Italy is shown in Figure S5 and Figure S6, respectively. Apart from *F. tricinctum* and *F. sporotrichioides*, the other *Fusarium* species were detected throughout the survey. However, the co-occurrence of all six species was never observed within a single grain sample. Most samples contained multiple *Fusarium* species, typically ranging from two to five, with combinations of four or five species being frequently observed in both macro-areas. (Figure 7). The strongest co-occurrence patterns were observed between *F. graminearum* and *F. avenaceum*, *F. graminearum* and *F. poae*, and *F. avenaceum* and *F. poae*.

### 2.4. Accumulation of Secondary Metabolites of Fusarium and Alternaria in Barley Grain

The fungal secondary metabolites (µg/kg) quantified by LC-MS/MS in barley samples collected from Northern and Central Italy in 2019 are summarized in Table 2, Table 3, Table 4, Table 5 and Table 6.

Since most of the secondary metabolites detected belonged to those produced by the fungal genera *Fusarium* and *Alternaria*, the results reported below will specifically refer to the secondary metabolites produced by the aforementioned genera. We would like to clarify that, although culmorin (CULM) and its derivatives (CULM-related metabolites), such as 15-hydroxyculmorin (15-HCULM), 15-hydroxyculmorone (15-HCULM-ONE) and 5-hydroxyculmorin (5-HCULM), are not chemically classified as trichothecenes, it was grouped with trichothecene metabolites because it is frequently co-produced by the same *Fusarium* species and commonly co-occur with DON and related trichothecenes in cereal grains.

Focusing on *Fusarium* secondary metabolites (Table 2), trichothecenes, CULM and CULM-related metabolites were detected at significantly higher levels in samples from Northern Italy compared with those from Central Italy (*p* = 0.002), as were depsipeptides (*p* = 0.007) and other *Fusarium* secondary metabolites (*p* = 0.004). Among *Fusarium* mycotoxins, only ZEN was detected at similar levels in samples from the two macro-areas (*p* = 0.62). Regarding *Alternaria* secondary metabolites (Table 2), the area of origin of the samples did not affect the total levels detected (*p* = 0.97).

Most trichothecenes, CULM and CULM-related metabolites (Table 3), were significantly more abundant (*p* < 0.05) in the Northern Italian area, including DON, 15-acetyldeoxynivalenol (15-ADON), deoxynivalenol-3-glucoside (DON-3-G), CULM, 15-HCULM, 15-HCULM-ONE, and 5-HCULM. In contrast, nivalenol (NIV), HT-2 toxin, and T-2 toxin showed similar levels in both macro-areas and occurred at generally low incidence. DON was the most frequent trichothecene, particularly in Northern Italy, where it exceeded the EU maximum level for unprocessed barley (1000 µg/kg, [32]) in three samples. DON derivatives (15-ADON, 3-ADON) and the modified form DON-3-G were detected almost exclusively in Northern Italy. CULM and CULM-related metabolites also showed high incidence and accumulation in Northern samples. NIV was detected with a very similar incidence and level in samples from the two areas, and a similar situation was also observed for HT-2 toxin and T-2 toxin, for which, overall, a very low incidence of positive samples was detected, and all were below the level admitted for barley [33].

For depsipeptides (Table 4), beauvericin (BEA) occurred only in a limited number of samples and showed no differences (*p* < 0.05) between macro-areas. In contrast, enniatins (ENNs) were detected in all barley samples, following the gradient ENB1 > ENB > ENA1 > ENB2 > ENA > ENB3, and accumulated in significantly higher levels in Northern Italy (*p* < 0.05).

Other *Fusarium* secondary metabolites such as aurofusarin (AUF), chrysogin (CHR), and moniliformin (MON) were also detected (Table 5). Among these, CHR was the only compound showing significant regional variation (*p* < 0.05), being more frequent in Northern Italy. AUF and MON were also more prevalent in Northern samples, although without significant differences in accumulation (*p* < 0.05).

In addition, seven *Alternaria* metabolites were identified: TeA, AOH, AME, tentoxin (TEN), altersetin (ALT), infectopyrone (INF), and macrosporin (MAC) (Table 6). Except for ALT, none showed significant differences in accumulation between macro-areas, although TeA, AOH, and TEN exhibited a higher incidence of positive samples in Northern Italy. For *Alternaria* toxins currently covered by the European Commission recommendation [34], which for cereals applies only to baby foods, TeA levels remained below the established limits, whereas AOH and AME exceeded them in some samples.

**Figure 8:**
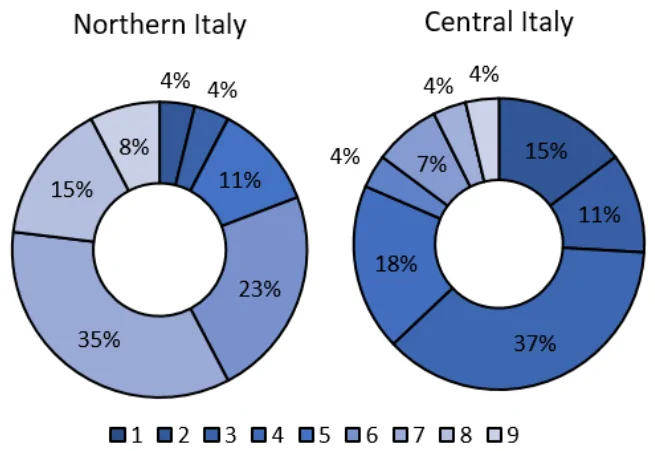
Percentage of barley samples collected in the macro-areas of Northern and Central Italy in 2019 showing co-presence (in a single sample) of 1, 2, 3, 4, 5, 6, 7, 8, or 9 *Fusarium* secondary metabolites categories [DON and derivatives (3A-DON, 15A-DON), NIV, CULM + CULM-related metabolites (CULM, 15-HCULM, 5-HCULM, 15-HCULM-ONE), T-2 + HT-2, BEA, ENNs, MON, AURO, CHRYSO] as determined by liquid chromatography tandem mass spectrometry (LC-MS/MS). See text for secondary metabolite abbreviations.

Co-occurrence patterns of *Fusarium* metabolites revealed a high degree of complexity, particularly in Northern Italy, where many samples contained five or six metabolite categories, and one sample showed all nine categories simultaneously (Figure 8). In Central Italy, most samples contained three or four categories, although one sample also showed all nine.

**Figure 9:**
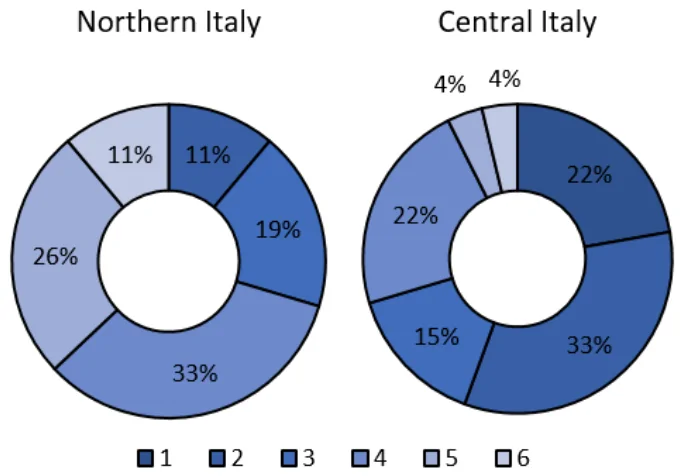
Percentage of barley samples collected in the macro-areas of Northern and Central Italy in 2019 showing co-presence (in a single sample) of 1, 2, 3, 4, 5 or 6 *Alternaria* secondary metabolites (TeA, AOH, AME, TEN, ALT, INF, and MAC) as determined by liquid chromatography tandem mass spectrometry (LC-MS/MS). See text for secondary metabolite abbreviations.

For *Alternaria* metabolites, Northern samples more frequently contained three compounds simultaneously, whereas Central samples more often contained two; one Central sample showed six *Alternaria* metabolites at once (Figure 9).

**Figure 10:**
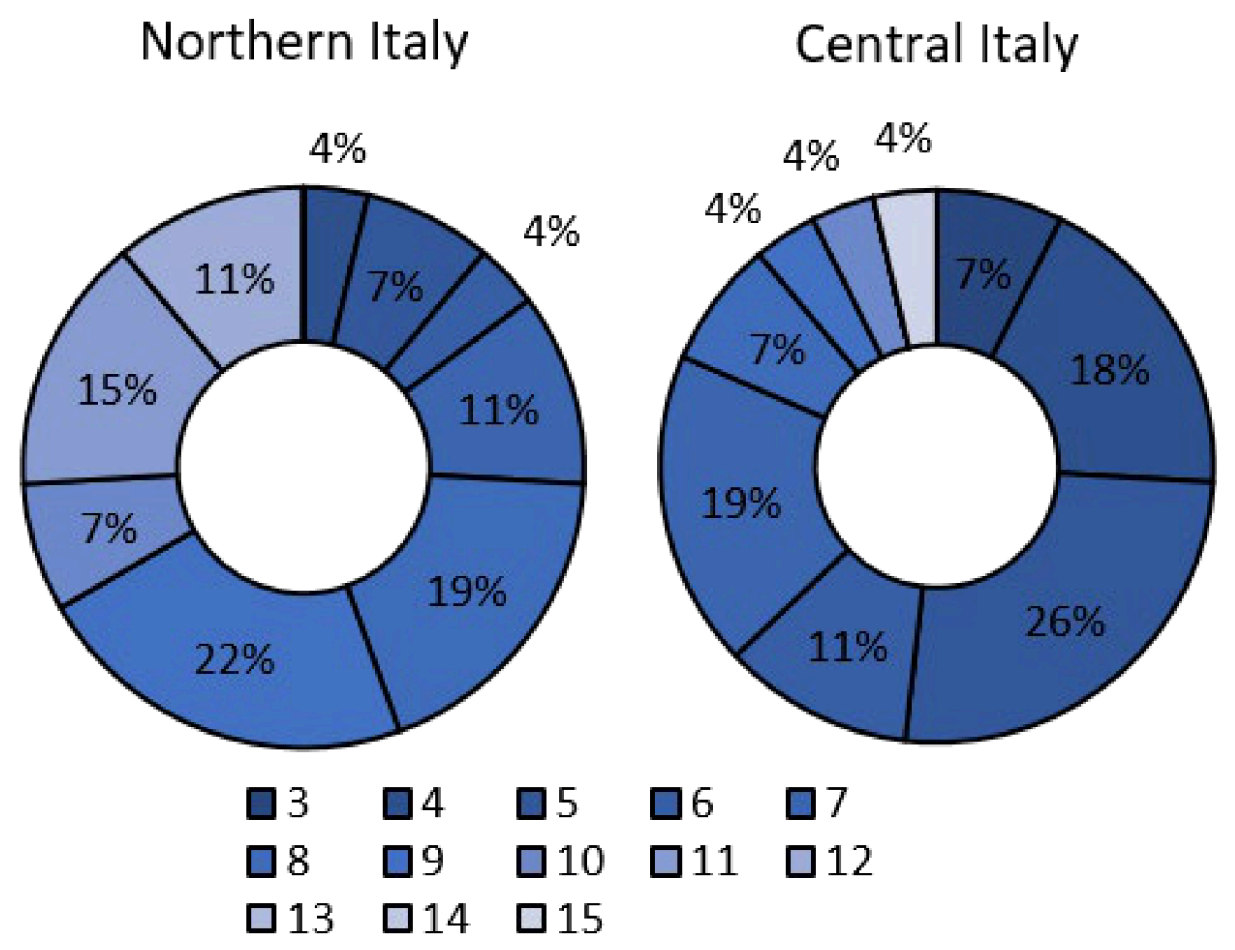
Percentage of barley samples collected in the macro-areas of Northern and Central Italy in 2019 showing co-presence (in a single sample) of 3, 4, 5, 6, 7, 8, 9, 10, 11, 12, 13, 14 or 15 *Fusarium* secondary metabolites [DON and derivatives (3A-DON, 15A-DON), NIV, CULM + CULM-related metabolites (CULM, 15-HCULM, 5-HCULM, 15-HCULM-ONE), T-2 + HT-2, BEA, ENNs, MON, AURO or CHRYSO] and *Alternaria* secondary metabolites (TeA, AOH, AME, TEN, ALT, INF or MAC) as determined by liquid chromatography tandem mass spectrometry (LC-MS/MS). See text for secondary metabolite abbreviations.

Finally, considering all metabolites produced by *Fusarium* and *Alternaria* together, no sample was free of these compounds (Figure 10). In Northern Italy, many samples contained eight or nine metabolites simultaneously, while in Central Italy combinations of four to seven metabolites were most common. Notably, one Central sample contained fifteen metabolites produced by the two genera.

### 2.5. Correlation Between Alternaria and Fusarium Accumulation and Their Secondary Metabolites

Correlations (r) between relative abundance detected by HTS of the *ITS* region of fungal ribosomal DNA of *Fusarium* or *Alternaria* and their secondary metabolites, determined by LC–MS/MS, are reported in Table 7, while the corresponding *p*-values are shown in Table S7. *Fusarium* abundance displayed a strong positive correlation with total *Fusarium* secondary metabolites, and similarly high correlations were observed with DON and its derivatives, as well as with CULM and CULM-related metabolites (CULM, 15-HCULM and 5-HCULM). A significant positive correlation was also observed between *Alternaria* relative abundance and total *Alternaria* secondary metabolites. Among individual compounds, TEN, INF, and MAC showed the highest and most significant positive correlations.

Additionally, correlations (r) between *Fusarium* species quantified by RT-qPCR and *Fusarium* secondary metabolites (determined by LC–MS/MS) are reported in Table 8, while the corresponding *p*-values are shown in Table S8. The strongest significant correlations are those between *F. graminearum* and DON, *F. avenaceum* and MON, and *F. langsethiae* and T-2 + HT-2 toxins.

## 3. Discussion

The present study aims to investigate, at the genus level, the fungal community associated with barley grain samples collected from two Italian cultivation macro-areas (Northern and Central Italy) using HTS of the *ITS* region of fungal ribosomal DNA, complemented by RT-qPCR analyses to determine the presence of the most relevant *Fusarium* species. In addition, LC-MS/MS analysis was performed to assess the occurrence of fungal secondary metabolites produced by *Fusarium* and *Alternaria*.

The present survey, using HTS of the *ITS* region of fungal ribosomal DNA, showed a prevalence of ascomycetes in the Northern macro-area, while basidiomycetes showed a prevalence in the Central macro-area. This suggests that the well-known climatic differences characterizing these two Italian macro-areas, where the Northern macro-area is more humid and cooler, and the Central macro-area is less humid and drier in comparison, may influence the distribution gradient of these two divisions. However, it should be emphasized that this study did not include specific analyses of climatic variables. Therefore, the differences observed between the two macro-areas are interpreted in light of the typical climatic characteristics generally associated with these regions of Italy. Also, other studies showed how they can be influenced by environmental parameters [52,53]. Within the *Ascomycota* division, six genera (*Alternaria*, *Aureobasidium*, *Cladosporium*, *Fusarium*, *Microdochium*, and *Pyrenophora*) were detected in this study. Additionally, other microorganisms belonging to the *Pleosporales* order (in addition to *Alternaria* and *Pyrenophora*) and the *Sclerotiniaceae* family were identified. Some of the detected ascomycetes, such as *Alternaria*, *Cladosporium*, *Fusarium*, *Microdochium*, and *Pyrenophora*, are involved in well-known barley diseases that affect mainly seeds but also other tissues such as spikes or leaves [21,29,30,31,32,33]. All these genera were frequently found in barley grain [6,13,14,16,20,22,23,24,25,54,55,56]. Within ascomycetes, the genus *Alternaria* was the most abundant in both Northern and Central Italy, with no significant differences between the two macro-areas. Also, in other surveys conducted in Italy, *Alternaria* was detected as the dominant genus in barley [22,23,24] and in wheat [35,36] mycobiota.

Interestingly, these surveys were conducted using a culture-dependent method, whereas the present one was conducted using a culture-independent method (HTS). However, regardless of the method used, *Alternaria* confirmed its dominance in Italy within a barley/wheat grain fungal community, as well as its ability to develop ubiquitously even within cultivation areas characterized by environmental differences. Also, in other cultivation areas around the world, culture-dependent or culture-independent methods of microorganisms’ detection showed predominance of *Alternaria* in barley grain [31,57,58,59,60,61]. *Alternaria* acts as a major causal agent of black point, a disease to which other fungal genera, such as *Cladosporium*, may also contribute [28,29]. *Cladosporium* was detected as the third and the second most abundant genus in Northern and Central macro-areas, respectively. Unlike *Alternaria*, *Cladosporium* abundance was influenced by the macro-area of cultivation, with the highest abundance in the Central macro-area in comparison to the Northern one. Previous studies [62,63] highlighted that *Cladosporium* incidence could increase under warmer climatic conditions, and this can explain the highest presence in the warmer macro-area of Central Italy in comparison to cooler Northern Italy. Although *Cladosporium* was commonly found in barley grain [6,14,16,19,25,55,56], the previous surveys conducted in Italy in barley and wheat grain detected *Cladosporium* only sporadically [22,23,24,38,39]. This could be because these surveys were conducted by culture-dependent methods, where *Cladosporium* may have been overgrown by other fungi with more rapid development, and its identification was difficult [59].

Unlike *Alternaria*, which did not show differences between the two cultivation areas, and *Cladosporium*, which showed a higher presence in the Central macro-area compared to the Northern macro-area, *Microdochium* and *Fusarium* exhibited a higher presence in the Northern macro-area than in the Central one. For *Fusarium* and *Microdochium*, this higher incidence in Northern Italy has been reported on wheat [35,64], and the present investigation confirms this trend also for barley grain. Moreover, while *Fusarium* has been widely reported in all previous analyses carried out in Italy on barley [22,23,24], *Microdochium* has been reported mainly on wheat [39,64]. However, in other areas of cultivation worldwide, *Microdochium* detections on barley grain have been reported several times together with *Fusarium* [56,65,66].

Similar to *Alternaria*, *Pyrenophora* did not show significant differences between Northern and Central Italy. This confirms what was observed in a previous survey conducted on Italian barley grain specifically targeting this pathogen [20]. Other investigations on Italian barley grain carried out using culture-dependent methods did not report the presence of *Pyrenophora* colonies [22,23,24]. A survey that reported the presence of *Pyrenophora* in Italian barley grains using a culture-dependent approach was specifically designed to detect this pathogen [20]. The differences observed in the present analysis can be attributed to the same reasons previously discussed for *Cladosporium*, as culture-dependent methods tend to limit the isolation of *Pyrenophora*, unless specific targeted analyses are performed [20].

Within the *Ascomycota* division, in addition to the well-known pathogenic genera described above, the presence of *Aureobasidium* was also detected. Microorganisms belonging to this genus have previously been reported in barley grain [13,25,56] and have been extensively studied for their ability to control FHB [67,68,69,70]. In the present study, a negative correlation between the genera *Fusarium* and *Aureobasidium* was observed, which is quite interesting in light of the existing evidence for its ability to act as a biological control agent (BCA). This negative association further supports the potential of *Aureobasidium* as a biological control resource that could be explored in future strategies aimed at reducing FHB pressure in barley.

Within the *Basidiomycota* division, nine genera (*Bullera* s.l., *Bulleromyces* s.l., *Cryptococcus* s.l., *Dioszegia*, *Exobasidium*, *Filobasidium*, *Hannaella*, *Puccinia*, *Sporobolomyces* s.l.) were detected in this study. Additionally, other microorganisms belonging to the *Tremellales* order (in addition to *Bullera* s.l., *Bulleromyces* s.l., *Cryptococcus* s.l., *Dioszegia*, *Filobasidium*, *Hannaella*, and *Sporobolomyces* s.l.) were identified.

Interestingly, none of these basidiomycetes were reported in previous culture-dependent studies of barley grain cultivated in Italy [22,23,24]. Notably, basidiomycetes are difficult to isolate using standard fungal isolation procedures and artificial media, due to factors such as slow growth in culture and the use of non-optimal isolation media. For these reasons, culture-independent methods enable the detection of microorganisms belonging to this division [71]. To confirm this statement, several studies conducted with culture-independent methods detected the presence of basidiomycetes in barley grain samples cultivated in different geographic areas [6,14,15,56].

Among basidiomycetes, *Cryptococcus* s.l. was by far the most frequently detected genus in both Northern and Central Italy. *Cryptococcus* was frequently detected in barley grains [6,16,25,72,73] as well as in wheat [12,74,75]. Until now, there is no evidence of its pathogenicity on cereal crops as well as in plants in general [76]. Conversely, this genus includes several strains classified as BCAs [77,78,79]. Some *Cryptococcus* species have been isolated from wheat flowers [80] and tested for their ability to control FHB, giving interesting positive results [81,82,83,84]. In this study, we observed a negative correlation between *Cryptococcus* and *Fusarium*, suggesting its potential action against these pathogens. In addition to *Fusarium*, our findings highlighted that *Cryptococcus* showed a negative correlation with other barley-pathogenic genera such as *Alternaria*, *Cladosporium*, *Pyrenophora*, and *Microdochium*, suggesting its potential against major ascomycete-related diseases. Its action against *Alternaria* is well-documented [85,86,87]. However, to our knowledge, the activity of *Cryptococcus* against *Cladosporium*, *Pyrenophora*, and *Microdochium* has not been reported. Nevertheless, caution is required when interpreting correlation data, as alternative scenarios cannot be excluded. Specifically, the observed correlations could also reflect the opposite situation to that described above, namely a scenario in which *Fusarium* or other pathogenic genera outcompete *Cryptococcus*, or where these genera exhibit opposite environmental preferences.

Following *Cryptococcus*, the other basidiomycete occurring with a notable incidence in the surveyed barley samples was *Dioszegia*. Unlike *Cryptococcus*, *Dioszegia* was present in Italian barley grains regardless of the cultivation macro-area. This genus has also been previously reported in barley [6,25,56,86,88,89], and similarly to *Cryptococcus*, no cases of pathogenicity in crops, including cereals, have been documented to date. In the present study, a negative correlation between *Dioszegia* and *Fusarium* was observed, suggesting that some species within this genus may serve as potential BCAs. However, the scientific literature available so far does not report any studies investigating the activity of *Dioszegia* against *Fusarium*. Therefore, experimental studies are required to determine whether these associations reflect true antagonistic interactions or simply different ecological preferences. *Dioszegia* is a genus of epiphytic basidiomycete yeasts commonly associated with the phyllosphere of plants. The genus was originally established based on *Dioszegia hungarica* [90], subsequently transferred to the genus *Cryptococcus* because of morphological similarities [91], and later reinstated following molecular phylogenetic studies demonstrating its clear distinction from *Cryptococcus* species [92,93]. Since its re-establishment, the genus has expanded considerably and currently comprises numerous described species distributed across a wide range of terrestrial environments [94,95,96,97]. Phylogenetic analyses have placed *Dioszegia* within the family *Bulleribasidiaceae* (order *Tremellales*) [98,99].

Among the basidiomycetes detected in the investigated sample, the only pathogen for barley was *Puccinia*. This genus was detected in very low amounts in some samples from Central Italy. Although this genus is known for causing rust in barley and other small-grain cereals, which is a typical foliar disease [100,101], in some cases of strong infection, it can also develop on the glumes of spikes [102], and this could explain its presence in grain.

The present study also highlighted the co-occurrence of at least eight ascomycetes and at least seven basidiomycetes within a single grain sample (from a single field of origin), showing that the investigated matrix provides an ecological niche that supports the simultaneous development of diverse fungi. Other studies conducted using culture-independent methods in barley [6,14,56], highlighted the co-occurrence of different microorganisms within a single ecological niche (e.g., a grain sample). This co-occurrence could create a network of interactions in which beneficial fungal communities can act against disease-causing fungi, already at levels of a single cereal seed, laying the foundation for a much deeper and potentially more effective use of beneficial microorganisms than has been achieved so far [103]. Generally, several yeast-like basidiomycetes detected in this study, including *Cryptococcus* and *Aureobasidium*, and possibly also *Dioszegia*, are potentially BCAs in cereal pathosystems, acting as antagonists of *Fusarium* spp. through mechanisms such as nutrient competition, rapid colonization of spike tissues and production of antifungal metabolites [67]. These documented antagonistic activities provide ecological support for the negative correlations observed in our dataset, suggesting that naturally occurring yeast communities may contribute to limiting pathogen development within barley grain. However, these co-occurrence patterns are inherently observational and cannot confirm a biocontrol activity. Therefore, such associations should be interpreted with caution, and targeted validation through *in vitro* co-culture assays and field inoculation trials will be required to determine whether these yeasts exert measurable antagonistic effects on cereal pathogens.

In this study, following an initial screening of the fungal genera present using *ITS*-based HTS, specific species of the *Fusarium* genus were identified and quantified through a targeted RT-qPCR assay. The investigation focused on eight *Fusarium* species potentially involved in barley FHB, considering their ability to biosynthesize secondary metabolites, some of which (mycotoxins) are of particular concern as harmful contaminants in human food and animal feed and are therefore regulated at the European level.

The FHB community associated with barley grain harvested in Northern Italy was dominated by *F. graminearum*, which showed the highest levels of accumulation in samples of this macro-area in comparison to those of the Central macro-area. Conversely, in the Central macro-area, the detected species did not show significant differences from each other. In addition to *F. graminearum*, *F. poae* also showed the highest levels of accumulation in barley grain samples of Northern Italy in comparison to those of Central Italy. Previous surveys conducted in barley grain in Italy were mainly focused on samples collected in a region of Central Italy (Umbria) using culturable methods [22,23,24]. These studies never reported *F. graminearum* as the most frequent species, confirming what was described in the present study in the Central macro-area with the RT-qPCR method. The highest presence of *F. graminearum* in the Northern macro-area was reported in Italy by surveys conducted in wheat grain [37,38], leading to the hypothesis that a similar trend may also occur in barley grain.

The previously mentioned surveys [22,23,24] detected a shift in the dominant species of the FHB community associated with barley grain in the Umbria region (Central Italy), which moved from *F. poae* and *F. avenaceum*, with a very low presence of *F. culmorum* and *F. proliferatum*. However, the present investigation, conducted across a wide area and using RT-qPCR assays, detected a considerable presence of *F. culmorum* and *F. proliferatum* both in Northern and Central Italy, highlighting the occurrence of these two species in barley grains in addition to wheat grain [37,38].

The present study highlighted the co-occurrence of more *Fusarium* species in barley grain collected in a single field. Even five different *Fusarium* species, out of the eight investigated, were simultaneously detected in some single barley samples (single field). Also, in this case, previous surveys conducted on barley grain from Central Italy detected, using culturable methods, the co-presence of multiple *Fusarium* species within a single sample [22,23,24]. However, the co-occurrence of *Fusarium* species within a single barley field was also detected in other cultivation areas worldwide [104,105], confirming that species of this genus can be simultaneously spread within a single barley field. This co-presence has also been reported at the level of a single wheat spike [51], and secondary metabolites produced by *Fusarium* species may play a crucial role in the possible relationship among *Fusarium* species within the same tissue [106].

*Fusarium* secondary metabolites showed a higher presence in the Northern macro-area; *Alternaria* secondary metabolites did not show differences between the two macro-areas investigated. This reflected the abundance of *Fusarium* and *Alternaria* detected by *ITS*-based HTS, which was higher in the Northern macro-area for *Fusarium* and similar in both macro-areas for *Alternaria*. This suggests the good reliability of the *ITS*-based HTS method for detecting specific mycotoxigenic microorganisms and, at the same time, as a reliable indicator of the possible presence of secondary metabolites produced by these microorganisms in the examined grain. A higher presence of *Fusarium* secondary metabolites in the Northern macro-area had already been highlighted in a survey conducted on wheat [35], indicating that, in general, even if not always [38], the Italian Northern macro-area represents, for both wheat and barley, a region with a higher risk of *Fusarium* secondary metabolite presence. Conversely, *Alternaria* showed a more homogeneous presence across different Italian cultivation areas, with, in some cases, a higher occurrence in non-Northern macro-areas [37,38].

Consistent with the overall pattern of *Fusarium* secondary metabolites, trichothecenes, particularly DON and its derivatives, as well as CULM and its derivatives, were highly prevalent in the surveyed samples and more common in the Northern macro-area. In contrast, other trichothecenes, such as HT-2 and T-2 toxins, were detected less frequently and did not differ between macro-areas. RT-qPCR analysis and correlation analysis with *Fusarium* secondary metabolites allowed us to hypothesize that DON accumulation was mainly a consequence of the presence of *F. graminearum*, which, being the most abundant species and particularly abundant in the Northern macro-area, also caused a higher incidence and higher accumulation levels of DON. Conversely, HT-2 and T-2 toxins were present at very low incidence due to the absence (*F. sporotrichioides*) or low presence (*F. langsethiae*), in both macro-areas, of the *Fusarium* species capable of producing them.

Previous surveys conducted in barley grains collected in Central Italy confirmed the lower presence of DON in this macro-area [22,23,24]. For this reason, Northern Italy can be defined as an area of barley cultivation with a higher potential for DON accumulation. To confirm this, in the present survey, three samples exceeded the limit (1000 µg/kg) admitted by the European Union for this matrix [31]. Conversely, none of the samples exceeded the limit (200 µg/kg) for the sum of HT-2 + T-2 toxins admitted by the European Union [32]. Variation in DON accumulation in barley grains collected from different cultivation areas was also detected in other countries [107]. In addition, a recent survey conducted in Northern Italy reported the presence of DON in barley grains together with the simultaneous absence of T-2 and HT-2 toxins [108]. Climatic factors such as temperature and moisture strongly influence the distribution of FHB pathogens and DON contamination across Europe, with cooler and wetter environments favoring *Fusarium* development and trichothecene accumulation. In general, FHB incidence is low or absent in the most southern regions of Italy and Spain, whereas more northern areas of Italy, Spain, Portugal, southern France and the Balkan Peninsula frequently report the occurrence of *F. graminearum* at cereal maturity together with DON [109].

Among depsipeptides, ENNs were detected in all analyzed samples, both in Northern and Central Italy, confirming the widespread occurrence of these compounds in cereals worldwide [110]. However, higher levels of accumulation were detected in the Northern macro-area. Previous surveys reported a higher incidence of ENNs in barley samples collected in Central Italy [24,111], as well as the presence of these compounds in barley samples collected from Northern Italy [109]. Surveys conducted in durum wheat showed a higher presence of ENNs in Northern Italy [37], although in another cultivation season, a higher presence of ENNs was found in Central Italy [38]. A high incidence of ENNs was also observed in barley grains cultivated in other countries [112].

In both surveyed macro-areas, the co-occurrence of different *Fusarium* secondary metabolites within a single barley grain sample was detected. This is a consequence of the previously discussed co-occurrence of multiple *Fusarium* species in single barley grain samples. The co-occurrence of *Fusarium* secondary metabolites had already been observed in previous surveys conducted in Central Italy [24]. The simultaneous presence of several *Fusarium* secondary metabolites within a single barley sample highlights the intricate network of potentially dangerous effects on human and animal health that can arise in such matrices. Above all, it is difficult to estimate the toxic effects deriving from the simultaneous presence of multiple secondary metabolites. However, several co-exposure studies have demonstrated synergistic toxic effects [113,114,115].

Consistent with the overall pattern of *Alternaria* secondary metabolites, TeA, AOH, AME, TEN, INF, and MAC showed individually no significant difference between the accumulation on barley grains collected in the two Italian macro-areas surveyed. The only exception is represented by ALT, which showed a higher level of accumulation in barley grain collected in the Northern macro-area. In addition, some *Alternaria* secondary metabolites showed low incidence (e.g., TeA, AOH, AME), while some others showed their presence in all analyzed samples (e.g., INF). *Alternaria* secondary metabolites were already detected in barley grains during previous surveys conducted in Central Italy [24,111] as well as in other cultivation areas worldwide [57,116,117].

As described for *Fusarium*, *Alternaria* secondary metabolites also showed co-occurrence within single barley grain samples. Co-exposure studies involving several *Alternaria* secondary metabolites demonstrated an increase in toxicity for combined *Alternaria* metabolites compared with their individual application [118]. In addition, the co-occurrence of *Fusarium* and *Alternaria* secondary metabolites also occurred within single barley grain samples. This co-occurrence had already been observed in individual barley grain samples collected in Central Italy [24], as well as in other cultivation areas. Synergistic toxic effects resulting from the combination of *Alternaria* and *Fusarium* secondary metabolites have been reported in co-exposure studies [119].

Overall, the differences observed between the two macro-areas have potential implications for crop monitoring and food safety. The higher prevalence of *F. graminearum* and trichothecenes in Northern Italy indicates that this region may require more intensive surveillance for FHB and mycotoxin contamination, particularly during seasons characterized by prolonged humidity during anthesis. Conversely, more homogeneous distribution of *Alternaria* across the two macro-areas suggests that risks associated with *Alternaria* should be monitored consistently throughout the country. The frequent co-occurrence of multiple *Fusarium* and *Alternaria* metabolites within individual grain samples further highlights the need for integrated monitoring strategies and preventive agronomic practices, including optimized harvest timing, crop residue management, and the use of cultivars with low susceptibility to fungal infection. These findings provide a biological basis for region-specific risk assessment and support the development of targeted approaches to crop health and food safety.

From a practical perspective, these results suggest that mycotoxin monitoring programs should adopt a multi-mycotoxin approach capable of simultaneously detecting metabolites from different fungal genera. In barley production systems, the observed geographic differences support the implementation of region-specific management strategies, including the selection of less susceptible cultivars and the optimization of agronomic practices according to local risk conditions. Moreover, the identification of fungal genera showing negative associations with potential pathogens highlights promising candidates for future biological control strategies.

However, the interpretation of the results should consider several limitations of this study, including the relatively small sample size, the focus on a single growing season, the lack of detailed information on agronomic practices and cultivated barley varieties, and the taxonomic constraints of *ITS*-based HTS, which limit fungal identification to the genus level. These factors may influence the generalizability of the observed patterns and should be considered when concluding.

## 4. Conclusions

In conclusion, this study provides insight into the mycobiota composition of barley grain samples harvested in two different macro-areas of Italy, as well as into the accumulation of *Alternaria* and *Fusarium* secondary metabolites.

This study combines HTS, RT-qPCR, and LC–MS/MS to characterize the mycobiota of Italian barley grain at multiple diagnostic levels. By simultaneously assessing fungal community structure, the abundance of key *Fusarium* species, and the accumulation of *Fusarium* and *Alternaria* secondary metabolites within the same samples, it provides an integrated overview of fungal biodiversity, pathogen occurrence, and mycotoxin contamination in barley grains from different Italian macro-areas.

Barley grain proved to be a niche for the development of both potentially pathogenic and non-pathogenic ascomycetes and basidiomycetes. Within the fungal community, mycotoxigenic genera such as *Alternaria* and *Fusarium* were frequently detected, and their secondary metabolites accumulated in the grain, often co-occurring within single barley samples originating from the same field. The complexity of the fungal network, together with the observed co-occurrence of their secondary metabolites, highlights the intricate interactions that can be established within a single grain sample from a single field. This complexity may have repercussions for human and animal health, given the potential synergistic effects of co-exposure to different mycotoxins.

Overall, the results of this study provide a useful basis for phytosanitary monitoring programs, support more accurate risk assessment of multi-mycotoxin contamination, and offer indications for the development of targeted crop management and future biological control strategies aimed at mitigating fungal pressure in barley, considering the different distributions of fungal genera across Italian macro-areas.

Future research should experimentally validate the interactions suggested by the correlation patterns observed in this study, particularly those involving potentially beneficial microorganisms and phytopathogenic fungi, to clarify their ecological roles and assess their potential use in biological control strategies.

## 5. Materials and Methods

### 5.1. Barley Grain Sampling

**Figure 11:**
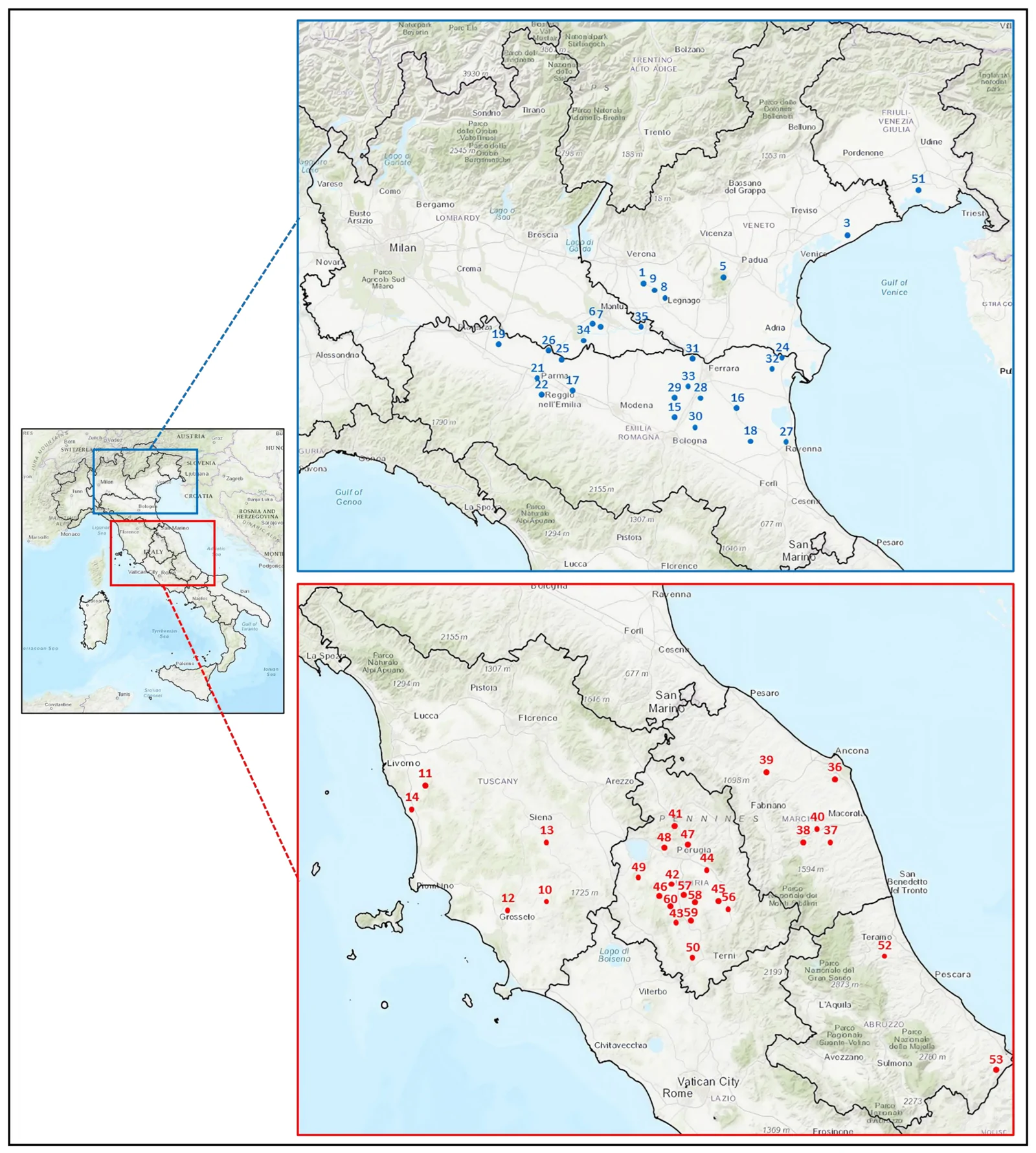
Geographic distribution of barley grain samples collected from two Italian macro-areas. The figure shows the entire country of Italy (**left**) and a zoomed-in view of the Northern and Central macro-areas (**right**). Each blue or red numbered point indicates the location of a collected sample, the details of which are reported in Table S9.

The current investigation was conducted on 54 barley samples collected in 2019 from eight distinct regions of Italy, grouped into two macro-areas (Northern and Central Italy), which typically differ in climatic conditions [45] (Figure 11). The 54 samples were evenly distributed, with 27 samples collected from each of the two macro-areas, allowing a direct comparison between the two macro-areas. The sampling strategy was designed to ensure the highest possible representativeness of the two Italian macro-areas. Detailed information for each sample is provided in Table S9. Agronomic information, including field management practices (e.g., fertilization and fungicide applications), was not available for the sampled fields.

Following harvest, each sample, approximately 1 kg in weight, was divided into three representative subsamples (100 g each): one designated for DNA mycobiota profiling using HTS *ITS* sequencing, another for DNA quantification of eight *Fusarium* species associated with FHB by RT-qPCR, and a third for quantification of fungal secondary metabolites in the grain through LC-MS/MS analysis.

### 5.2. Total DNA Extraction from Barley Samples

A single DNA extraction protocol was employed for both the mycobiota analysis (via HTS) and the quantification of DNA from eight *Fusarium* species commonly associated with FHB, using RT-qPCR.

Briefly, for each of the two purposes, about 100 g of each barley sample was finely ground with a GM200 blender (Retsch, Haan, Germany), and 4 g of ground grains were used for total DNA (barley DNA and total mycobiota DNA) extraction using the method previously described [120]. The isolated DNA was left at 4 °C overnight and then stored at −20 °C until use.

### 5.3. DNA-Based Mycobiota Determination by High-Throughput Sequencing of the Fungal Internal Transcribed Spacer Region

The *ITS2* region was amplified with primers f*ITS*7 (5′-TGTGARTCATCGAATCTTTG-3′) [121] and *ITS*4 (5′-TCCTCCGCTTATTGATATGC-3′) [122]. Both primers were tagged with an 8 bp unique barcode [123]. The PCR reactions contained 200 µM dNTP, 750 µM MgCl2, 1.25 units of DreamTaq polymerase (Thermo Fisher Scientific, Waltham, MA, USA), 0.5 µM of forward primer and 0.3 µM of reverse primer. The PCR conditions were 5 min at 95 °C and 27–35 cycles (depending on the amount of PCR product evaluated by gel electrophoresis) of 30 s at 95 °C, 30 s at 57 °C and 30 s at 72 °C. Samples were run in duplicates, which were pooled and purified with AMPure beads (Beckman Coulter Inc., Brea, CA, USA). Purified PCR products were pooled and concentrated with the E.Z.N.A. Omega cycle pure kit (Omega Bio-tek, Norcross, GA, USA). Libraries for Illumina sequencing were prepared from 50 ng of amplicon DNA using the Smarter ThruPLEX DNA-seq Kit (Takara Bio Inc., Kusatsu, Japan) with HT dual indexes (Takara Bio Inc.). The library preparation was performed according to the manufacturer’s instructions. A sequencing library for the phage PhiX was included as 10% spike-in in the sequencing run. Sequencing was performed together with two other libraries in one flow cell on the Illumina MiSeq system (Illumina Inc., San Diego, CA, USA) (2 × 300 bp) using the v3 sequencing chemistry. Demultiplexing, merging of paired-end reads, quality filtering, and clustering were performed with the SCATA pipeline (https://scata.mykopat.slu.se, URL (accessed on 11 May 2021) [124]. Raw data (6 423 202 reads) were filtered on length (>200 bp), quality (mean quality > Q20, base quality > Q10), and the presence of both primers (2 mismatches allowed). After quality filtering, 15.6% of reads remained, which were clustered at 98.5% similarity in SCATA, producing 214 OTUs represented by >10 reads. The clustering threshold was selected based on previous experience with cereal mycobiome data and published literature [125]. Taxonomic classification of OTUs was performed with the RDP classifier [126] with the UNITE training dataset (2014-07-08). Ten non-fungal clusters were removed from the dataset.

### 5.4. DNA Quantification of Eight Fusarium Species in Barley Grains by Real-Time Quantitative PCR

For the *Fusarium* DNA quantification by RT-qPCR, the concentration of total extracted DNA was assessed with NanoDrop One (Thermo Fisher Scientific) and was adjusted to 25 ng/µL. To establish standard curves for RT-qPCR assays, DNA was extracted from pure fungal cultures of eight selected *Fusarium* species. Detailed information about the reference strains is provided in Table S10 [22,35,111,127,128]. All selected *Fusarium* species were cultured on potato dextrose agar (PDA, Biolife Italiana, Milan, Italy) for one week at 22 °C in darkness. Mycelium was harvested using a spatula, transferred to 2 mL plastic tubes (Eppendorf), and freeze-dried using a lyophilizer (Heto Powder Dry LL3000, Thermo Fisher Scientific). Subsequently, one steel bead (Qiagen, Hilden, Germany) was added to each tube, and the fungal tissue was finely ground using a MM 400 (Retsch) at a frequency of 25 Hz for 6 min. Fungal DNA extraction followed the protocol previously outlined [127]. The same procedure was applied to DNA extracted from barley grains to generate the standard curve. DNA quality and concentration were assessed using Nanodrop One (Thermo Fisher Scientific). Dilution series ranging from 20 ng to 0.02 pg of fungal strain DNA and from 200 ng to 2 pg of barley DNA, with a dilution factor of 10, were employed to generate standard curves in each RT-qPCR set, with two technical replicates per assay. Species-specific primers listed in Table S11 were utilized for RT-qPCR analyses [129,130]. Standard curve parameters, including line equations, R^2^ values, reaction efficiencies, and limits of detection (LOD), were determined following the methodology previously described [120]. RT-qPCR assays were conducted using a CFX96 real-time PCR detection system (Bio-Rad, Hercules, CA, USA). Annealing temperatures (ranging from 55 °C to 65 °C) were experimentally adjusted for each primer pair to optimize RT-qPCR reaction conditions. The RT-qPCR mixture used for both standard curves and barley grain analysis comprised a total reaction volume of 12 µL, containing 2.5 µL of total DNA, 6 µL of 2X SYBR Select Master Mix for CFX (Applied Biosystem, Foster City, CA, USA), 1.5 μL of 2 μM of each primer, and 0.5 μL of sterile DNase-free water (Thermo Fisher Scientific). The amplification cycle included initial denaturation at 95 °C for 10 min, followed by 45 cycles of denaturation at 95 °C for 15 s, annealing at specific temperatures for 1 min, heating at 95 °C for 10 s, cooling at 60 °C, and a final increase to 95 °C at 0.5 °C every 5 s with fluorescence measurement. A dissociation curve was included at the end of the RT-qPCR program to monitor the presence of primer-dimers and/or non-specific amplification products. Each assay utilized two replicates per barley sample. The fungal biomass of each investigated *Fusarium* species, potentially present in the barley grain, was expressed as the ratio of detected fungal DNA (pg) to total barley grain DNA (ng).

### 5.5. Detection and Quantification in Barley Grains of Fungal Secondary Metabolites Produced by Fusarium and Alternaria by LC-MS/MS

Five grams of ground samples were extracted and subsequently analyzed for secondary metabolites as previously described [131]. The extraction was performed in 50 mL polypropylene tubes (Sarstedt, Nümbrecht, Germany) using 20 mL of extraction solvent (acetonitrile/water/acetic acid 79:20:1, *v*/*v*/*v*). The mixture was incubated for 90 min at 25 °C while shaking on a GFL 3017 rotary shaker (GFL, Burgwedel, Germany), and aliquots of the raw extracts were pipetted into autosampler vials and diluted 1 + 1 with dilution solvent (acetonitrile/water/acetic acid 20:79:1, *v*/*v*/*v*). If metabolite concentration exceeded the highest calibration standard, extracts were further diluted (1 + 49 and/or 1 + 999) and re-analyzed. Analysis was carried out on a QTrap 5500 MS/MS system (Sciex, Framingham, MA, USA) equipped with a TurboV ElectroSpray Ionisation (ESI) source, coupled to a 1290 series Infinity II UHPLC system (Agilent Technologies, Waldbronn, Germany). A Gemini C18 column, 150 × 4.6 mm i.d., 5 μm particle size, equipped with a C18 security guard cartridge, 4 × 3 mm i.d. (both Phenomenex, Torrance, CA, USA) was used in the gradient elution mode using two acidified methanol/water mixtures containing 1% acetic acid and 5 mM ammonium acetate. In scheduled Multiple Reaction Monitoring Mode, two MS/MS transitions were acquired per analyte, except in the case of moniliformin and 3-nitroropionic acid, since each yielded only one product ion. Identification was confirmed by ensuring that the ion ratio agreed with the relative values of the corresponding authentic standard within 30%. A retention time criterion of ± 0.03 min was applied. Quantification was performed using external calibration with a serial dilution of a multi-analyte stock solution. To prepare this multi-analyte stock solution, individual reference compounds were dissolved (mostly in pure acetonitrile, a few in acetonitrile-water 1/1 or pure methanol) and combined in >50 intermediate mixes of 10 compounds each, which were subsequently mixed. Results were corrected for apparent recoveries obtained by spiking experiments. Limits of detection and of quantification were determined following the EURACHEM guide [131,132]. The method’s accuracy is verified routinely on a routine basis through participation in a proficiency testing scheme organized by BIPEA (Gennevilliers, France), achieving a 96% rate of satisfactory z-scores (−2 < z < 2) for the >2300 results submitted to date.

### 5.6. Statistical Analysis

All datasets related to: (1) fungal microorganisms associated with barley grain samples detected by high-throughput *ITS* sequencing; (2) the eight main *Fusarium* species associated with FHB accumulated in barley grain samples and detected by RT-qPCR; and (3) secondary metabolites produced by *Fusarium* and *Alternaria* species and accumulated in barley grain samples, detected by LC–MS/MS, were subjected to one-way analysis of variance (ANOVA). Comparisons were performed either between the two macro-areas (Northern and Central Italy) or among different samples within the same macro-area. When ANOVA indicated statistically significant differences (*p* < 0.05), pairwise comparisons were carried out using Tukey’s honestly significant difference (HSD) post hoc test, which was used to account for multiple pairwise comparisons among means. No data transformations were applied. Statistical analyses were performed using the DSAASTAT Macro (version 1.0192; [133]) for Excel^®^. Finally, correlations between: (1) potentially pathogenic and non-potentially pathogenic fungal genera detected by high-throughput *ITS* sequencing; (2) the accumulation of eight *Fusarium* species detected by RT-qPCR and the secondary metabolites they produced, detected by LC–MS/MS; and (3) *Alternaria* and *Fusarium* fungal genera detected by high-throughput *ITS* sequencing and the secondary metabolites they produced, detected by LC–MS/MS, were evaluated using the Pearson correlation coefficient (r), followed by Student’s t-test, using Excel^®^ (Tables S4, S7 and S8).

## Figures and Tables

**Table 1:** Correlations (r) among barley grain mycobiota. Colour intensity reflects correlation strength (−1 to +1; red = negative, green = positive).

	*Alt*	*Cla*	*Mic*	*Pyr*	*Fus*	Other *Ple*	*Aur*	*Scl*	Unk *Asc*	Other *Asc*	*Cry*	*Dio*	*Bul*	*Han*	Other *Tre*	*Fil*	*Exo*	*Spo*	*Bull*	*Puc*	Unk *Bas*	Other *Bas*
*Alt* ^a^	1																					
*Cla* ^b^	0.35	1																				
*Mic* ^c^	−0.31	−0.23	1																			
*Pyr* ^d^	−0.04	0.12	−0.09	1																		
*Fus* ^e^	−0.21	−0.09	0.40	−0.11	1																	
Other *Ple* ^f^	0.57	0.12	0.09	−0.15	0.09	1																
*Aur* ^g^	0.11	0.34	−0.47	0.05	−0.22	−0.28	1															
*Scl* ^h^	−0.15	0.14	−0.08	0.18	0.09	−0.15	−0.07	1														
Unk *Asc* ^i^	0.07	−0.17	0.30	0.45	0.21	0.12	−0.41	−0.01	1													
Other *Asc* ^j^	0.24	0.15	0.02	−0.04	0.27	0.19	0.00	0.08	0.17	1												
*Cry* ^k^	−0.53	−0.33	−0.31	−0.36	−0.24	−0.39	0.10	−0.08	−0.46	−0.50	1											
*Dio* ^l^	−0.09	−0.29	0.01	−0.02	−0.15	−0.15	0.05	−0.03	−0.13	0.32	−0.21	1										
*Bull* ^m^	−0.41	−0.11	0.14	−0.14	0.27	−0.15	−0.03	0.39	−0.17	0.01	0.11	0.08	1									
*Han* ^n^	−0.26	−0.23	0.13	−0.09	0.19	−0.11	−0.08	0.25	−0.02	0.12	0.03	0.18	0.50	1								
Other *Tre* ^o^	0.15	−0.07	−0.27	0.00	−0.09	−0.31	0.15	0.07	−0.12	−0.08	0.12	−0.02	−0.18	−0.02	1							
*Fil* ^p^	0.29	0.28	−0.42	0.20	−0.20	−0.04	0.65	−0.18	−0.22	−0.03	−0.06	0.02	−0.32	−0.30	0.27	1						
*Exo* ^q^	−0.18	−0.19	0.53	0.05	0.24	0.04	−0.31	0.16	0.14	0.28	−0.35	0.37	0.26	0.26	−0.13	−0.30	1					
*Spo* ^r^	−0.29	0.08	−0.26	0.09	−0.10	−0.34	0.26	0.37	−0.23	−0.26	0.30	−0.11	0.57	0.06	−0.04	0.00	−0.06	1				
*Bul* ^s^	−0.10	−0.27	0.16	−0.02	0.06	0.16	−0.21	−0.06	0.13	0.07	−0.13	0.33	0.34	0.09	−0.13	−0.20	0.37	−0.04	1			
*Puc* ^t^	−0.07	0.02	−0.23	−0.14	−0.22	−0.07	0.03	0.00	−0.19	0.18	0.06	0.46	0.06	0.03	−0.13	−0.07	−0.20	0.05	0.16	1		
Unk *Bas* ^u^	−0.30	−0.14	−0.44	−0.19	−0.34	−0.47	0.28	0.06	−0.42	−0.16	0.52	0.24	0.30	0.07	0.09	0.07	−0.37	0.46	0.03	0.55	1	
Other *Bas* ^v^	−0.19	−0.20	−0.05	0.50	−0.17	−0.19	0.05	−0.06	0.35	0.18	−0.09	0.27	−0.10	−0.02	−0.05	−0.07	0.07	−0.06	0.12	0.14	0.03	1

^a^ *Alternaria*; ^b^ *Cladosporium*; ^c^ *Microdochium*; ^d^ *Pyrenophora*; ^e^ *Fusarium*; ^f^ Other *Pleosporales*; ^g^ *Aureobasidium*; ^h^ *Sclerotiniaceae*; ^i^ Unknown ascomycetes; ^j^ Other ascomycetes; ^k^ *Cryptococcus sensu lato*; ^l^ *Dioszegia*; ^m^ *Bulleromyces sensu lato*; ^n^ *Hannaella*; ^o^ Other *Tremellales*; ^p^ *Filobasidium*; ^q^ *Exobasidium*; ^r^ *Sporobolomyces sensu lato*; ^s^ *Bullera sensu lato*; ^t^ *Puccinia*; ^u^ Unknown basidiomycetes; ^v^ Other basidiomycetes.

**Table 2:** Amounts of Fusarium and Alternaria secondary metabolites detected by liquid chromatography–tandem mass spectrometry (LC–MS/MS) in barley grain samples collected in the macro-areas of Northern and Central Italy in 2019. Different letters in the multiple comparison test column indicate statistically significant differences (*p* < 0.05) between the Northern and Central macro-areas according to Tukey’s HSD test.

Categories of Secondary Metabolites	Positive Samples (n = 27)	Incidence Positive Samples (%)	Average Positive Samples (µg/kg) ± Standard Error		Total Average (µg/kg) ± Standard Error		Multiple Comparison Test (Total Average)
*Fusarium* secondary metabolites							
Trichothecenes, culmorin and culmorin-related compounds							
Northern Italy	25	93	642	±182	595	±171	a
Central Italy	15	56	71.3	±20.1	39.6	±13.0	b
Zearalenone							
Northern Italy	6	22	8.00	±5.52	1.85	±1.31	a
Central Italy	2	7	14.4	±8.82	0.21	±0.21	a
Depsipeptides							
Northern Italy	27	100	90.4	±16.1	90.4	±16.1	a
Central Italy	27	100	44.1	±18.7	44.1	±18.7	b
Other *Fusarium* secondary metabolites							
Northern Italy	25	93	1536	±421	1422	±398	a
Central Italy	21	78	223	±100	182	±82.5	b
*Alternaria* secondary metabolites							
Northern Italy	27	100	262	±57.0	262	±57.0	a
Central Italy	27	100	265	±53.4	265	±53.4	a

**Table 3:** Amounts of trichothecenes and culmorin-related compounds detected by liquid chromatography–tandem mass spectrometry (LC–MS/MS) in barley grain samples collected in the macro-areas of Northern and Central Italy in 2019. Different letters in the multiple comparison test column indicate statistically significant differences (*p* < 0.05) between the Northern and Central macro-areas according to Tukey’s HSD test.

Secondary Metabolites	Positive Samples (n = 27)	Incidence Positive Samples (%)	Average Positive Samples (µg/kg) ± Standard Error		Total Average (µg/kg) ± Standard Error		Max Value (µg/kg)	Multiple Comparison Test (Total Average)
*Fusarium* secondary metabolites								
Trichothecenes, culmorin and culmorin-related compounds								
Deoxynivalenol								
Northern Italy	25	93	493	±130	456	±127	3183	a
Central Italy	11	41	62.2	±17.5	25.3	±9.17	193.5	b
15-Acetyldeoxynivalenol								
Northern Italy	9	33	166	±40.5	55.2	±22.0	445	a
Central Italy	0	0	0.00	-	0.00	-	0.00	b
3-Acetyldeoxynivalenol								
Northern Italy	9	33	49.3	±17.2	16.4	±7.16	173	a
Central Italy	1	4	33.4	-	1.24	±1.24	33.4	a
Nivalenol								
Northern Italy	7	26	29.3	±6.11	7.59	±2.92	63.2	a
Central Italy	5	19	31.9	±2.78	5.91	±2.48	37.5	a
Deoxynivalenol-3-glucoside								
Northern Italy	19	70	76.6	±21.3	56.8	±16.6	410	a
Central Italy	5	19	22.8	±6.32	4.2	±2.04	46.0	b
Culmorin								
Northern Italy	21	78	408	±88.5	317	±86.3	1662	a
Central Italy	7	26	167	±27.8	43.2	±18.5	398	b
15-Hydroxyculmorin								
Northern Italy	23	85	437	±124	373	±127	2699	a
Central Italy	7	26	185	±56.7	48.1	±21.1	447	b
15-Hydroxyculmorone								
Northern Italy	8	30	54.1	±15.2	16.0	±6.48	140	a
Central Italy	1	4	10.4	-	0.38	±0.38	10.4	b
5-Hydroxyculmorin								
Northern Italy	15	56	1072	±307	596	±198	4716	a
Central Italy	3	11	347	±87.8	38.6	±22.9	497	b
HT-2 toxin								
Northern Italy	1	4	28.6	-	1.06	(±1.06)	28.6	a
Central Italy	3	11	25.1	±2.72	2.78	(±1.72)	40.6	a
T-2 toxin								
Northern Italy	3	11	8.87	±1.48	0.99	(±0.56)	11.5	a
Central Italy	1	4	2.71	-	0.10	(±0.10)	2.71	a

**Table 4:** Amounts of depsipeptides detected by liquid chromatography–tandem mass spectrometry (LC–MS/MS) in barley grain samples collected in the macro-areas of Northern and Central Italy in 2019. Different letters in the multiple comparison test column indicate statistically significant differences (*p* < 0.05) between the Northern and Central macro-areas according to Tukey’s HSD test.

Secondary Metabolites	Positive Samples (n = 27)	Incidence Positive Samples (%)	Average Positive Samples (µg/kg) ± Standard Error		Total Average (µg/kg) ± Standard Error		Max Value (µg/kg)	Multiple Comparison Test (Total Average)
*Fusarium* secondary metabolites								
Depsipeptides								
Beauvericin								
Northern Italy	16	59	1.29	±0.43	0.76	±0.36	5.45	a
Central Italy	9	33	0.81	±0.46	0.27	±0.21	4.43	a
Total Enniatins								
Northern Italy	27	100	99.8	±21.0	99.8	±21.0	437	a
Central Italy	27	100	33.6	±11.1	33.6	±11.1	276	b
Enniatin A								
Northern Italy	25	93	0.73	±0.38	0.68	±0.37	7.48	a
Central Italy	12	44	0.32	±0.15	0.15	±0.10	1.58	a
Enniatin A1								
Northern Italy	26	96	7.15	±2.67	6.89	±2.64	44.4	a
Central Italy	22	81	2.43	±1.30	1.98	±1.19	20.6	b
Enniatin B								
Northern Italy	27	100	31.5	±7.00	31.5	±7.00	170	a
Central Italy	27	100	11.3	±3.01	11.3	±3.01	72.1	b
Enniatin B1								
Northern Italy	27	100	55.1	±11.9	55.1	±11.9	218	a
Central Italy	26	96	19.19	±6.99	18.5	±6.0	172	b
Enniatin B2								
Northern Italy	23	85	6.65	±1.69	5.66	±1.51	38.5	a
Central Italy	21	78	2.19	±0.50	1.70	±0.43	9.93	b
Enniatin B3								
Northern Italy	18	67	0.04	±0.007	0.03	±0.006	0.08	a
Central Italy	13	48	0.02	±0.002	0.01	±0.002	0.04	b

**Table 5:** Amounts of other *Fusarium* secondary metabolites detected by liquid chromatography–tandem mass spectrometry (LC–MS/MS) in barley grain samples collected in the macro-areas of Northern and Central Italy in 2019. Different letters in the multiple comparison test column indicate statistically significant differences (*p* < 0.05) between the Northern and Central macro-areas according to Tukey’s HSD test.

Secondary Metabolites	Positive Samples (n = 27)	Incidence Positive Samples (%)	Average Positive Samples (µg/kg) ± Standard Error		Total Average (µg/kg) ± Standard Error		Max Value (µg/kg)	Multiple Comparison Test (Total Average)
*Fusarium* secondary metabolites								
Others								
Aurofusarin								
Northern Italy	12	44	67.3	±15.6	29.9	±9.43	189	a
Central Italy	7	26	62.0	±29.5	16.1	±8.96	235	a
Chrysogin								
Northern Italy	24	89	61.1	±11.9	54.3	±11.2	273	a
Central Italy	8	30	34.0	±9.64	10.1	±3.97	75.1	b
Moniliformin								
Northern Italy	12	44	67.3	±15.6	29.9	±9.8	189	a
Central Italy	7	26	62.0	±22.5	16.1	±9.3	235	a

**Table 6:** Amounts of *Alternaria* secondary metabolites detected by liquid chromatography–tandem mass spectrometry (LC–MS/MS) in barley grain samples collected in the macro-areas of Northern and Central Italy in 2019. Different letters in the multiple comparison test column indicate statistically significant differences (*p* < 0.05) between the Northern and Central macro-areas according to Tukey’s HSD test.

Secondary Metabolites	Positive Samples (n = 27)	Incidence Positive Samples (%)	Average Positive Samples (µg/kg) ± Standard Error		Total Average (µg/kg) ± Standard Error		Max Value (µg/kg)	Multiple Comparison Test (Total Average)
*Alternaria* secondary metabolites								
Tenuazonic acid								
Northern Italy	6	22	169	±55.3	37.6	±15.3	384	a
Central Italy	7	26	129	±46.2	33.5	±32.4	325	a
Alternariol								
Northern Italy	3	11	3.53	±2.81	0.39	±0.33	9.15	a
Central Italy	2	7	2.26	±0.92	0.17	±0.13	3.18	a
Alternariol monomethyl ether								
Northern Italy	1	4	5.51	-	0.20	±0.20	5.51	a
Central Italy	1	4	0.26	-	0.01	±0.01	0.26	a
Altersetin								
Northern Italy	11	41	2.85	±0.82	1.16	±0.42	10.70	a
Central Italy	3	11	1.81	-	0.20	±0.11	1.81	b
Tentoxin								
Northern Italy	23	85	5.62	±0.78	4.79	±0.77	15.8	a
Central Italy	14	52	6.82	±0.27	3.54	±1.50	39.2	a
Infectopyrone								
Northern Italy	27	100	221	±49.5	221	±49.5	1393	a
Central Italy	27	100	224	±43.6	224	±43.6	639	a
Macrosporin								
Northern Italy	12	44	0.37	±0.09	0.17	±0.05	1.16	a
Central Italy	17	63	0.738	±0.26	0.46	±0.18	4.77	a

**Table 7:** Correlations (r) between *Fusarium* and *Alternaria* relative abundance (*ITS* high-throughput sequencing) and their secondary metabolites (LC-MS/MS) in barley grains collected in Italy in 2019. Colour intensity indicates correlation strength (−1 to +1; red = negative, green = positive).

Secondary Metabolites	r
Total *Fusarium* secondary metabolites	0.76
Deoxynivalenol	0.76
15-Acetyldeoxynivalenol	0.71
3-Acetyldeoxynivalenol	0.74
Deoxynivalenol-3-glucoside	0.74
Nivalenol	0.28
Culmorin	0.73
15-Hydroxyculmorin	0.71
15-Hydroxyculmoron	0.70
5-Hydroxyculmorin	0.75
HT-2 toxin	0.19
T-2 toxin	0.33
Beauvericin	0.36
Enniatins	0.30
Aurofusarin	0.07
Chrysogin	0.71
Moniliformin	0.68
Total *Alternaria* secondary metabolites	0.34
Tenuazonic acid	0.17
Alternariol	−0.01
Alternariol monomethyl ether	−0.06
Altersetin	−0.02
Tentoxin	0.46
Infectopyrone	0.33
Macrosporin	0.31

**Table 8:** Correlations (r) between *Fusarium* species abundance (RT-qPCR) and their secondary metabolites (LC-MS/MS) in barley grains collected in Italy in 2019. Colour intensity indicates correlation strength (−1 to +1; red = negative, green = positive).

*Fusarium* Species	Secondary Metabolites	r
*F. graminearum*	Deoxynivalenol	0.87
	Nivalenol	0.24
*F. culmorum*	Deoxynivalenol	−0.004
	Nivalenol	0.10
*F. avenaceum*	Enniatins	−0.02
	Moniliformin	0.34
*F. poae*	Nivalenol	0.10
*F. langsethiae*	T2 + HT2	0.51

## Data Availability

The original contributions presented in this study are included in the article/Supplementary Materials. Further inquiries can be directed to the corresponding author.

## Supplementary Materials

The following supporting information can be downloaded at: https://www.mdpi.com/article/10.3390/toxins18090363/s1. Table S1: Relative abundance (%) of ascomycetes and basidiomycetes in barley grain samples in the macro-areas of Northern and Central Italy in 2019, as determined by high-throughput sequencing of the *ITS* region of fungal ribosomal DNA. In the multiple comparison test column, different letters indicate a statistically significant difference (*p* < 0.05), determined using Tukey’s Honestly Significant Difference (HSD) test; Table S2: Relative abundance (%) of fungal microorganisms belonging to the *Ascomycota* division in barley grain samples in the macro-areas of Northern and Central Italy in 2019, as determined by high-throughput sequencing of the *ITS* region of fungal ribosomal DNA. In the multiple comparison test column, different letters indicate a statistically significant difference (*p* < 0.05), determined using Tukey’s Honestly Significant Difference (HSD) test; Table S3: Relative abundance (%) of fungal microorganisms belonging to the *Basidiomycota* division in barley grain samples in the macro-areas of Northern and Central Italy in 2019, as determined by high-throughput sequencing of the *ITS* region of fungal ribosomal DNA. In the multiple comparison test column, different letters indicate a statistically significant difference (*p* < 0.05), determined using Tukey’s Honestly Significant Difference (HSD) test; Table S4: *p*-values, calculated using Student’s t-test, for correlations (r) between potentially pathogenic fungal genera affecting barley and other components of the barley mycobiota, identified in grains collected in Italy in 2019 by high-throughput sequencing of the *ITS* region of fungal ribosomal DNA; Table S5. R^2^ values and efficiency of the RT-qPCR reactions; Table S6: DNA amount (pg of each analyzed fungal species DNA/ng of barley) of eight *Fusarium* species in barley grain samples in the macro-areas of Northern and Central Italy in 2019, as determined by quantitative real-time polymerase chain reaction (RT-qPCR). In the multiple comparison test column, different letters indicate a statistically significant difference (*p* < 0.05), determined using Tukey’s Honestly Significant Difference (HSD) test; Table S7: *p*-values, calculated using Student’s t-test, for correlations (r) between *Fusarium* or *Alternaria* relative abundance (detected by high-throughput sequencing of the *ITS* region of fungal ribosomal DNA) and *Fusarium* or *Alternaria* secondary metabolites (detected by LC-MS/MS) and accumulated in barley grains collected in Italy in 2019; Table S8: *p*-values, calculated using Student’s t-test, for correlations (r) between eight *Fusarium* species (detected by RT-qPCR) and *Fusarium* secondary metabolites typically produced by that species (detected by LC-MS/MS) accumulated in barley grains collected in Italy in 2019; Table S9: Detailed information on the barley samples collected in Italy in 2019; Table S10. *Fusarium* strains used to obtain standard curves for real-time quantitative PCR (RT-qPCR) assays; Table S11. Primers used in quantitative real-time polymerase chain reaction (RT-qPCR) assays; Figure S1: Relative abundance (%) of fungal microorganisms belonging to the *Ascomycota* division in each single barley grain sample collected in the macro-areas of Northern Italy in 2019, as determined by high-throughput sequencing of the *ITS* region of fungal ribosomal DNA; Figure S2: Relative abundance (%) of fungal microorganisms belonging to the *Ascomycota* division in each single barley grain sample collected in the macro-areas of Central Italy in 2019, as determined by high-throughput sequencing of the *ITS* region of fungal ribosomal DNA; Figure S3: Relative abundance (%) of fungal microorganisms belonging to the *Basidiomycota* division in each single barley grain sample collected in the macro-areas of Northern Italy in 2019, as determined by high-throughput sequencing of the *ITS* region of fungal ribosomal DNA; Figure S4. Relative abundance (%) of fungal microorganisms belonging to the *Basidiomycota* division in each single barley grain sample collected in the macro-areas of Central Italy in 2019, as determined by high-throughput sequencing of the *ITS* region of fungal ribosomal DNA. Figure S5: DNA amount (pg of each analyzed fungal species DNA/ng of barley) of eight Fusarium species in each single barley grain sample collected in the macro areas of Northern Italy in 2019, as determined by quantitative real-time polymerase chain reaction (qPCR). Figure S6: DNA amount (pg of each analyzed fungal species DNA/ng of barley) of eight Fusarium species in each single barley grain sample collected in the macro areas of Central Italy in 2019, as determined by quantitative real-time polymerase chain reaction (qPCR).
