# Supplementary material for: High-Throughput Sequencing Study of the Mycobiota of Barley Grain Collected in Northern and Central Italy and Accumulation of Fungal Secondary Metabolites
Source: Toxins (Basel). 2026 Aug 25;18(9):363. doi: 10.3390/toxins18090363 (PMC13611532; doi:10.3390/toxins18090363)
Supplement: Supplementary file 1 [file toxins-18-00363-s001.zip › toxins-4416025-supplementary.pdf]

Supplementary Materials: High-throughput sequencing study of the mycobiota of barley grain collected in Northern and Central Italy and accumulation of fungal secondary metabolites

Lorenzo Covarelli, Giovanni Beccari, Emilio Balducci, Ida Karlsson, Paula Persson, Hanna Friberg, Michael Sulyok, Antonio Prodi, Massimo Montanari and Francesco Tini

Table S1. Relative abundance (%) of ascomycetes and basidiomycetes in barley grain samples in the macro areas of Northern and Central Italy in 2019, as determined by high-throughput sequencing of the *ITS* region of fungal ribosomal DNA. In the multiple comparison test column, different letters indicate a statistically significant difference ($p<0.05$), determined using Tukey's Honestly Significant Difference (HSD) test.

Macro area	Division	Relative abundance (%)	Multiple comparison test
Northern Italy	Ascomycetes	65.8	a
	Basidiomycetes	34.2	b
Central Italy	Ascomycetes	51.1	a
	Basidiomycetes	48.8	a
**Division**	**Area**	**Relative abundance (%)**	**Multiple comparison test**
Ascomycetes	Northern Italy	65.8	a
	Central Italy	51.1	b
Basidiomycetes	Northern Italy	34.1	b
	Central Italy	48.8	a

Table S2. Relative abundance (%) of fungal microorganisms belonging to the *Ascomycota* division in barley grain samples in the macro areas of Northern and Central Italy in 2019, as determined by high-throughput sequencing of the *ITS* region of fungal ribosomal DNA. In the multiple comparison test column, different letters indicate a statistically significant difference (p<0.05), determined using Tukey's Honestly Significant Difference (HSD) test.

Area	Ascomycetes	Relative abundance (%)	Multiple comparison test
Northern Italy	*Alternaria*	22.7	a
	Aureobasidium	0.4	f
	Cladosporium	8.4	c
	Fusarium	5.2	cde
	Microdochium	13.2	b
	Other ascomycetes	1.3	ef
	Other *Pleosporales*	2.3	ef
	Pyrenophora	3.6	def
	Sclerotiniaceae	1.2	ef
	Unknown ascomycetes	7.3	cd
Central Italy	*Alternaria*	25.9	a
	Aureobasidium	1.5	c
	Cladosporium	11.4	b
	Fusarium	1.2	c
	Microdochium	2.3	c
	Other ascomycetes	1.0	c
	Other *Pleosporales*	1.8	c
	Pyrenophora	1.9	c
	Sclerotiniaceae	0.7	c
	Unknown ascomycetes	3.6	c
**Ascomycetes**	**Area**	**Relative abundance (%)**	**Multiple comparison test**
Alternaria	Northern Italy	22.7	a
	Central Italy	25.9	a
Aureobasidium	Northern Italy	0.4	b
	Central Italy	1.5	a
Cladosporium	Northern Italy	8.4	b
	Central Italy	11.4	a
Fusarium	Northern Italy	5.2	a
	Central Italy	1.2	b
Microdochium	Northern Italy	13.2	a
	Central Italy	2.3	b
Other ascomycetes	Northern Italy	1.3	a
	Central Italy	1.0	a
Other *Pleosporales*	Northern Italy	2.3	a
	Central Italy	1.8	a
Pyrenophora	Northern Italy	3.6	a
	Central Italy	1.9	a
Sclerotiniaceae	Northern Italy	1.2	a
	Central Italy	0.7	a
Unknown ascomycetes	Northern Italy	7.3	a
	Central Italy	3.6	b

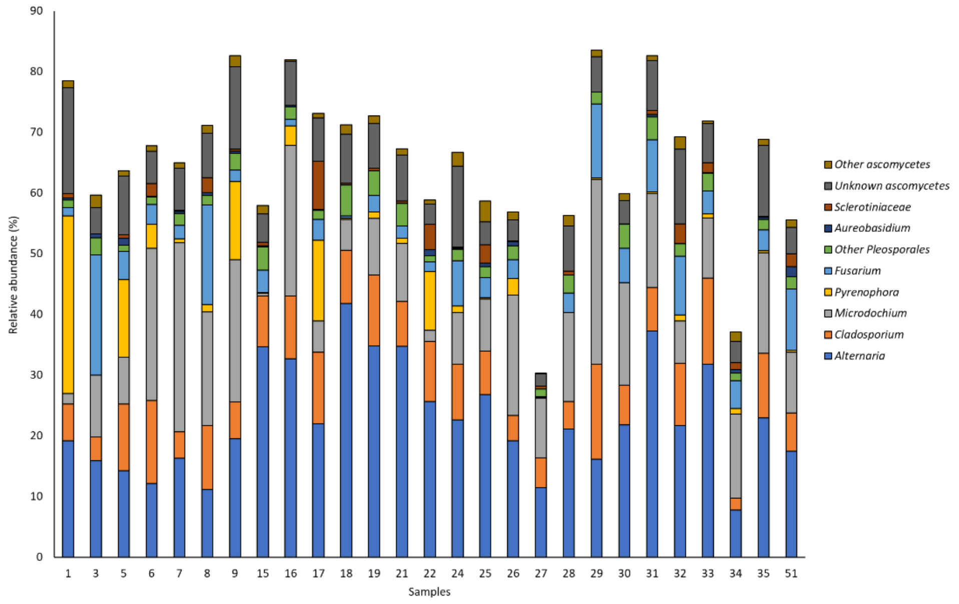

Figure S1. Relative abundance (%) of fungal microorganisms belonging to the *Ascomycota* division in each single barley grain sample collected in the macro areas of Northern Italy in 2019, as determined by high-throughput sequencing of the *ITS* region of fungal ribosomal DNA.

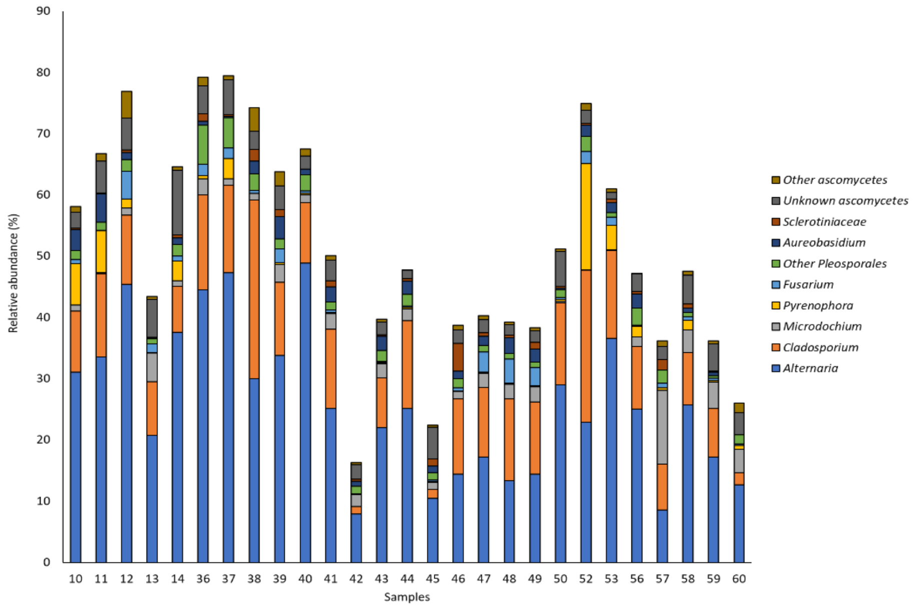

Figure S2. Relative abundance (%) of fungal microorganisms belonging to the *Ascomycota* division in each single barley grain sample collected in the macro areas of Central Italy in 2019, as determined by high-throughput sequencing of the *ITS* region of fungal ribosomal DNA.

Table S3. Relative abundance (%) of fungal microorganisms belonging to the *Basidiomycota* division in barley grain samples in the macro areas of Northern and Central Italy in 2019, as determined by high-throughput sequencing of the *ITS* region of fungal ribosomal DNA. In the multiple comparison test column, different letters indicate a statistically significant difference ($p < 0.05$), determined using Tukey's Honestly Significant Difference (HSD) test.

Area	Basidiomycetes	Relative abundance (%)	Multiple comparison test
Northern Italy	*Bullera sensu lato*	0.2	c
	Bulleromyces sensu lato	0.7	c
	Cryptococcus sensu lato	22.9	a
	Dioszegia	7.8	b
	Exobasidium	0.4	c
	Filobasidium	0.1	c
	Hannaella	0.4	c
	Other basidiomycetes	0.2	c
	Puccinia	0.0	c
	Sporobolomyces sensu lato	0.1	c
	Other *Tremellales*	0.2	c
	Unknown basidiomycetes	1.5	c
Central Italy	*Bullera sensu lato*	0.1	c
	Bulleromyces sensu lato	0.5	c
	Cryptococcus sensu lato	35.5	a
	Dioszegia	7.7	b
	Exobasidium	0.1	c
	Filobasidium	0.3	c
	Hannaella	0.2	c
	Other basidiomycetes	0.2	c
	Puccinia	0.0	c
	Sporobolomyces sensu lato	0.1	c
	Other *Tremellales*	0.3	c
	Unknown basidiomycetes	3.6	bc
Basidiomycetes	**Area**	**Relative abundance (%)**	**Multiple comparison test**
Bullera sensu lato	Northern Italy	0.2	a
	Central Italy	0.1	b
Bulleromyces sensu lato	Northern Italy	0.7	a
	Central Italy	0.5	a
Cryptococcus sensu lato	Northern Italy	22.9	b
	Central Italy	35.5	a
Dioszegia	Northern Italy	7.8	a
	Central Italy	7.7	a
Exobasidium	Northern Italy	0.4	a
	Central Italy	0.1	b
Filobasidium	Northern Italy	0.1	b
	Central Italy	0.3	a
Hannaella	Northern Italy	0.4	a
	Central Italy	0.2	a
Other basidiomycetes	Northern Italy	0.2	a
	Central Italy	0.2	a
Puccinia	Northern Italy	0.0	b
	Central Italy	0.0	a
Sporobolomyces sensu lato	Northern Italy	0.1	a
	Central Italy	0.1	a
Other *Tremellales*	Northern Italy	0.2	b
	Central Italy	0.3	a
Unknown basidiomycetes	Northern Italy	1.5	a
	Central Italy	3.6	b

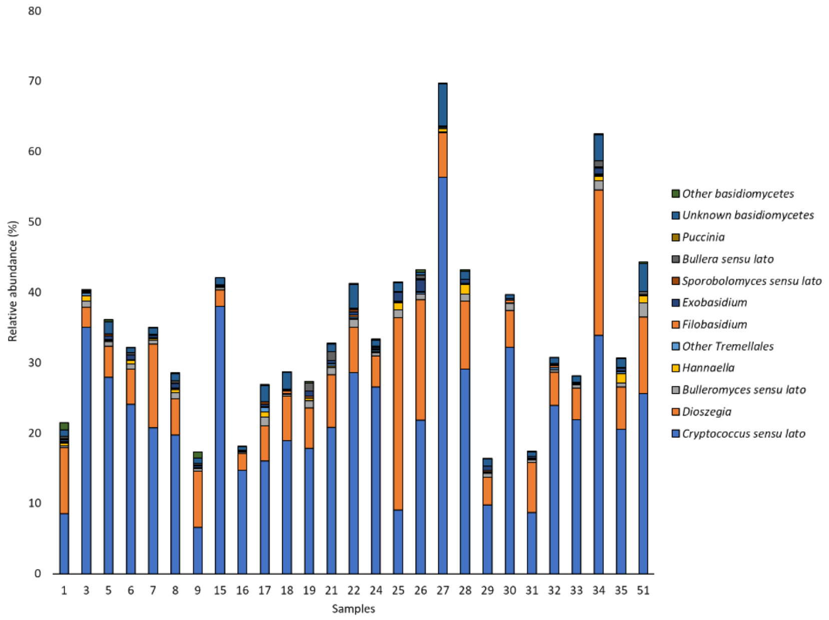

Figure S3. Relative abundance (%) of fungal microorganisms belonging to the *Basidiomycota* division in each single barley grain sample collected in the macro areas of Northern Italy in 2019, as determined by high-throughput sequencing of the *ITS* region of fungal ribosomal DNA.

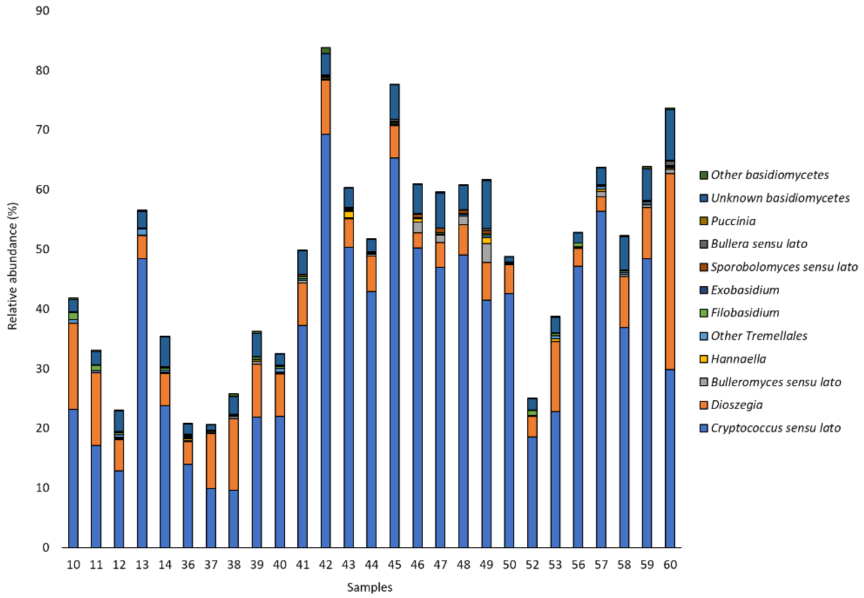

Figure S4. Relative abundance (%) of fungal microorganisms belonging to the *Basidiomycota* division in each single barley grain sample collected in the macro areas of Central Italy in 2019, as determined by high-throughput sequencing of the *ITS* region of fungal ribosomal DNA.

Table S4. P-values, calculated using Student's t-test, for correlations (r) between potentially pathogenic fungal genera affecting barley and other components of the barley mycobiota, identified in grains collected in Italy in 2019 by high-throughput sequencing of the *ITS* region of fungal ribosomal DNA.

Fungal genera/categories	*Alternaria*	*Cladosporium*	*Microdochium*	*Pyrenophora*	*Fusarium*
Alternaria					
Cladosporium	1.0×10^{-14}				
Microdochium	7.4×10^{-24}	1.0×10^{-1}			
Pyrenophora	2.7×10^{-24}	1.0×10^{-10}	3.1×10^{-4}		
Fusarium	4.7×10^{-25}	1.0×10^{-11}	4.2×10^{-4}	6.3×10^{-1}	
Puccinia	1.4×10^{-31}	1.0×10^{-26}	4.3×10^{-10}	3.3×10^{-4}	7.9×10^{-8}
Other *Pleosporales*	2.0×10^{-28}	8.8×10^{-20}	2.6×10^{-6}	4.0×10^{-1}	6.1×10^{-2}
Aureobasidium	4.2×10^{-30}	1.5×10^{-23}	2.9×10^{-8}	1.9×10^{-2}	1.5×10^{-4}
Sclerotiniaceae	6.4×10^{-30}	1.4×10^{-22}	4.0×10^{-8}	2.4×10^{-2}	2.8×10^{-4}
Unkown ascomycetes	2.9×10^{-22}	6.0×10^{-7}	6.6×10^{-2}	2.3×10^{-3}	2.2×10^{-3}
Other ascomycetes	6.6×10^{-30}	8.7×10^{-23}	5.5×10^{-8}	3.3×10^{-2}	4.1×10^{-4}
Cryptococcus sensu lato	6×10^{-2}	5.5×10^{-14}	1.2×10^{-14}	4.8×10^{-21}	3.1×10^{-21}
Dioszegia	4.5×10^{-17}	4.2×10^{-2}	9.8×10^{-1}	9.2×10^{-6}	6.3×10^{-6}
Bulleromyces sensu lato	1.1×10^{-30}	1.2×10^{-24}	6.1×10^{-9}	4.9×10^{-3}	1.0×10^{-5}
Hannaella	3.6×10^{-31}	8.7×10^{-26}	1.4×10^{-9}	1.2×10^{-3}	7.8×10^{-7}
Other *Tremellales*	2.8×10^{-31}	4.5×10^{-26}	1.0×10^{-9}	8.4×10^{-4}	4.1×10^{-7}
Filobasidium	2.8×10^{-30}	5.3×10^{-26}	1.1×10^{-9}	9.2×10^{-4}	4.9×10^{-7}
Exobasidium	3.2×10^{-31}	6.8×10^{-26}	1.3×10^{-9}	1.0×10^{-3}	6.1×10^{-7}
Sporobolomyces sensu lato	2.0×10^{-31}	2.2×10^{-26}	6.8×10^{-10}	5.4×10^{-4}	1.9×10^{-7}
Bullera sensu lato	2.3×10^{-31}	3.1×10^{-26}	8.1×10^{-10}	6.4×10^{-4}	2.6×10^{-7}
Unkown basidiomycetes	2.0×10^{-27}	6.8×10^{-17}	1.8×10^{-5}	8.1×10^{-1}	3.0×10^{-1}
Other basidiomycetes	2.4×10^{-31}	3.5×10^{-26}	8.8×10^{-10}	7.1×10^{-4}	3.0×10^{-7}

Table S5. R2 values and efficiency of the qPCR reactions.

***Fusarium* species**	R^2	**Efficiency (%)**
F. avenaceum	1.00	97
F. culmorum	0.99	100
F. graminearum	1.00	98
F. langsethiae	0.99	100
F. poae	0.99	100
F. proliferatum	1.00	99
F. sporotrichioides	1.00	99
F. tricinctum	1.00	100
Barley	0.99	98

Table S6. DNA amount (pg of each analyzed fungal species DNA/ng of barley) of eight *Fusarium* species in barley grain samples in the macro areas of Northern and Central Italy in 2019, as determined by quantitative real-time polymerase chain reaction (qPCR). In the multiple comparison test column, different letters indicate a statistically significant difference ($p < 0.05$), determined using Tukey's Honestly Significant Difference (HSD) test.

Area	*Fusarium* species	pg fungal DNA/ng wheat DNA	Multiple comparison test
Northern Italy	*F. graminearum*	0.889	a
	F. tricinctum	0.000	b
	F. culmorum	0.098	b
	F. langsethiae	0.004	b
	F. poae	0.161	b
	F. sporotrichioides	0.000	b
	F. proliferatum	0.021	b
	F. avenaceum	0.014	b
Central Italy	*F. graminearum*	0.034	a
	F. tricinctum	0.000	a
	F. culmorum	0.088	a
	F. langsethiae	0.004	a
	F. poae	0.063	a
	F. sporotrichioides	0.000	a
	F. proliferatum	0.037	a
	F. avenaceum	0.012	a
***Fusarium* species**	**Area**	**pg fungal DNA/ng wheat DNA**	**Multiple comparison test**
F. graminearum	Northern Italy	0.889	a
	Central Italy	0.034	b
F. culmorum	Northern Italy	0.098	a
	Central Italy	0.088	a
F. avenaceum	Northern Italy	0.014	a
	Central Italy	0.012	a
F. poae	Northern Italy	0.161	a
	Central Italy	0.063	b
F. tricinctum	Northern Italy	0.000	a
	Central Italy	0.000	a
F. sporotrichioides	Northern Italy	0.000	a
	Central Italy	0.000	a
F. langsethiae	Northern Italy	0.004	a
	Central Italy	0.004	a
F. proliferatum	Northern Italy	0.021	a
	Central Italy	0.037	a

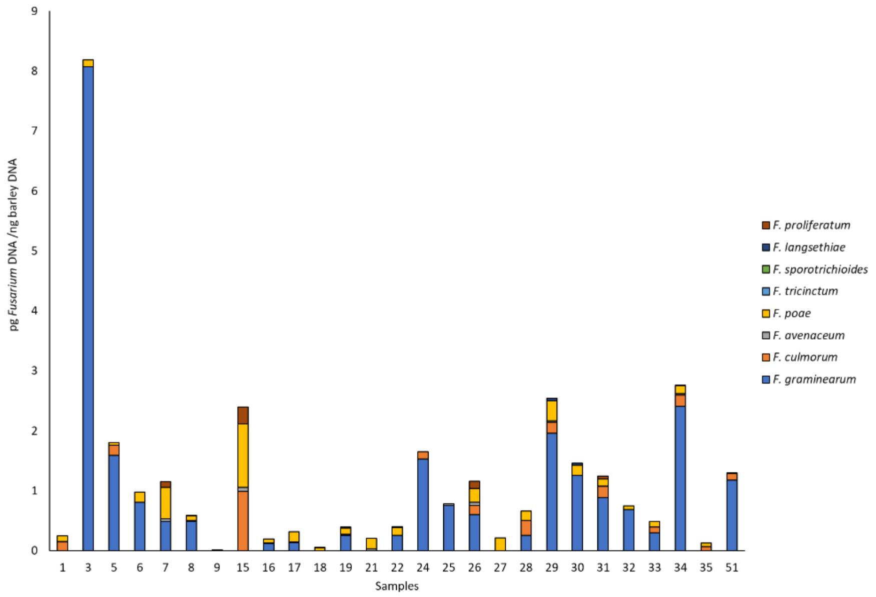

Figure S5. DNA amount (pg of each analyzed fungal species DNA/ng of barley) of eight *Fusarium* species in each single barley grain sample collected in the macro areas of Northern Italy in 2019, as determined by quantitative real-time polymerase chain reaction (qPCR).

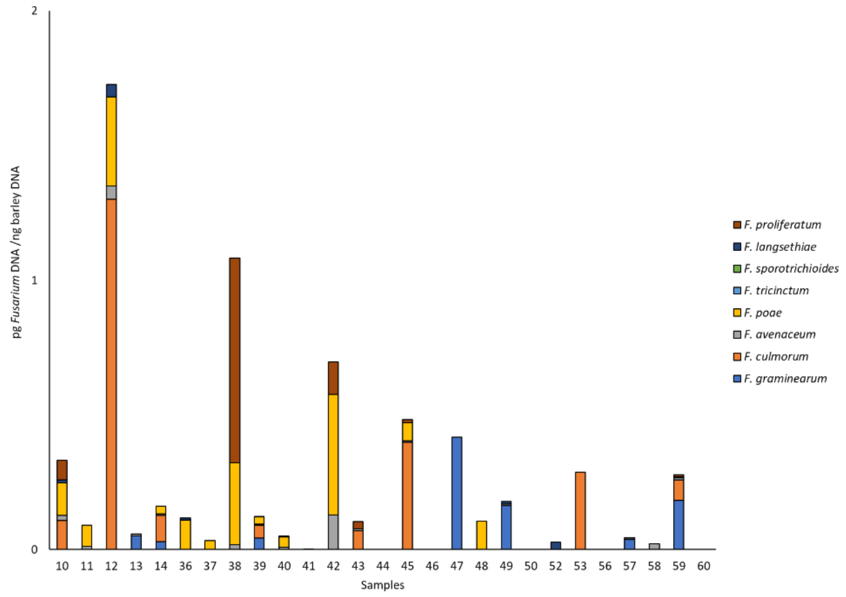

Figure S6. DNA amount (pg of each analyzed fungal species DNA/ng of barley) of eight *Fusarium* species in each single barley grain sample collected in the macro areas of Central Italy in 2019, as determined by quantitative real-time polymerase chain reaction (qPCR).

Table S7. P-values, calculated using Student's t-test, for correlations (r) between *Fusarium* or *Alternaria* relative abundance (detected by high-throughput sequencing of the *ITS* region of fungal ribosomal DNA) and *Fusarium* or *Alternaria* secondary metabolites (detected by LC-MS/MS) and accumulated in barley grains collected in Italy in 2019.

**Secondary metabolites**	**p-value**
Total *Fusarium* secondary metabolites	2.75×10^{-4}
Deoxynivalenol	9.25×10^{-4}
15-Acetyldeoxynivalenol	2.39×10^{-2}
3-Acetyldeoxynivalenol	1.38×10^{-1}
Deoxynivalenol-3-glucoside	3.23×10^{-3}
Nivalenol	7.38×10^{-2}
Culmorin	7.96×10^{-5}
15-Hydroxyculmorin	9.00×10^{-4}
15-Hydroxyculmoron	1.46×10^{-1}
5-Hydroxyculmorin	3.71×10^{-3}
HT-2 toxin	2.77×10^{-1}
T-2 toxin	4.74×10^{-5}
Beauvericin	9.89×10^{-6}
Enniatins	2.08×10^{-6}
Aurofusarin	4.30×10^{-2}
Chrysogin	1.29×10^{-6}
Moniliformin	3.06×10^{-3}
Total *Alternaria* secondary metabolites	1.22 x 10^{-8}
Tenuazonic acid	3.40 x 10^{-1}
Alternariol	6.11×10^{-31}
Alternariol monomethyl ether	2.37×10^{-31}
Altersetin	3.09×10^{-30}
Tentoxin	1.67×10^{-21}
Infectopyrone	2.18×10^{-8}
Macrosporin	4.49×10^{-31}

Table S8. P-values, calculated using Student's t-test, for correlations (r) between eight *Fusarium* species (detected by qPCR) and *Fusarium* secondary metabolites typically produced by that species (detected by LC-MS/MS) accumulated in barley grains collected in Italy in 2019.

***Fusarium* species**	**Secondary metabolites**	**p-value**
F. graminearum	Deoxynivalenol	8.13×10^{-4}
	Nivalenol	1.34×10^{-3}
F. culmorum	Deoxynivalenol	7.99×10^{-4}
	Nivalenol	7.00 x 10^{-4}
F. avenaceum	Enniatins	7.03×10^{-7}
	Moniliformin	6.20×10^{-4}
F. poae	Nivalenol	7.06×10^{-4}
F. langsethiae	T2+HT2	3.52×10^{-2}

Table S9. Detailed information on the barley samples collected in Italy in 2019.

Sample ID	Area of Italy	Region	Province	Location	Cultivar	Row	Main destination
1	Northern	Veneto	Verona	Isola Rizza	Planet	Two	Malting
3	Northern	Veneto	Venezia	Eraclea	Laureate	Two	Malting
5	Northern	Veneto	Padova	Torreglia	Planet	Two	Malting
6	Northern	Veneto	Rovigo	Arquà Polesine	Planet	Two	Malting
7	Northern	Veneto	Rovigo	Arquà Polesine	Meseta	Two	Feed
8	Northern	Veneto	Verona	Angiari	Concerto	Two	Malting
9	Northern	Veneto	Verona	Roverchiara	Planet	Two	Malting
10	Central	Tuscany	Grosseto	Roccalbegna	Lutece	Six	Feed
11	Central	Tuscany	Pisa	Santa Luce	Lutece	Six	Feed
12	Central	Tuscany	Grosseto	Grosseto	Aldebaran	Six	Feed
13	Central	Tuscany	Siena	Monteroni D'Arbia	Aldebaran	Six	Feed
14	Central	Tuscany	Livorno	Castiglioncello	Lutece	Six	Feed
15	Northern	Emilia Romagna	Bologna	San Pietro in Casale	Sandra	Two	Food
16	Northern	Emilia Romagna	Ferrara	Argenta	Sandra	Two	Food
17	Northern	Emilia Romagna	Reggio Emilia	Sant'Ilario D'Enza	Planet	Two	Malting
18	Northern	Emilia Romagna	Ravenna	Fusignano	Concerto	Two	Malting
19	Northern	Emilia Romagna	Parma	Busseto	Planet	Two	Malting
21	Northern	Emilia Romagna	Parma	Parma	Planet	Two	Malting
22	Northern	Emilia Romagna	Parma	Montechiarugolo	Planet	Two	Malting
24	Northern	Emilia Romagna	Ferrara	Mesola	Sandra	Two	Food
25	Northern	Emilia Romagna	Reggio Emilia	Poviglio	Lutece	Six	Feed
26	Northern	Emilia Romagna	Reggio Emilia	Poviglio	Lutece	Six	Feed
27	Northern	Emilia Romagna	Ravenna	Ravenna	Sandra	Two	Food
28	Northern	Emilia Romagna	Bologna	Malalbergo	Sandra	Two	Food
29	Northern	Emilia Romagna	Bologna	Argelato	Sandra	Two	Food
30	Northern	Emilia Romagna	Bologna	Granarolo Dell'Emilia	Rondo	Six	Food
31	Northern	Emilia Romagna	Ferrara	Bondeno	Rondo	Six	Food
32	Northern	Emilia Romagna	Ferrara	Mesola	Sandra	Two	Food
33	Northern	Emilia Romagna	Ferrara	Poggio Renatico	Cometa	Two	Feed
34	Northern	Lombardy	Mantova	Sabbioneta	Concerto	Two	Malting
35	Northern	Lombardy	Mantova	Castel D'Ario	Meseta	Two	Feed
36	Central	The Marche	Ancona	Osimo	Digersano	Two	Food
37	Central	The Marche	Macerata	Mogliano	Digersano	Two	Food
38	Central	The Marche	Ancona	Caldarola	Digersano	Two	Food
39	Central	The Marche	Ancona	Serra De' Conti	Concerto	Two	Malting
40	Central	The Marche	Macerata	Tolentino	Azzurro	Six	Feed
41	Central	Umbria	Perugia	Umbertide	Sandra	Two	Food
42	Central	Umbria	Perugia	Marsciano	Sandra	Two	Food
43	Central	Umbria	Perugia	Todi	Sandra	Two	Food
44	Central	Umbria	Perugia	Bastia Umbra	Sandra	Two	Food
45	Central	Umbria	Perugia	Montefalco	Sandra	Two	Food
46	Central	Umbria	Perugia	Marsciano	Quench	Two	Malting
47	Central	Umbria	Perugia	Perugia	Quench	Two	Malting
48	Central	Umbria	Perugia	Corciano	Quench	Two	Malting
49	Central	Umbria	Perugia	Panicale	Quench	Two	Malting
50	Central	Umbria	Terni	Montecastrilli	Callas	Two	Feed
51	Northern	Friuli Venezia Giulia	Udine	Marano Lagunare	Planet	Two	Malting
52	Central	Abruzzo	Teramo	Mosciano	Planet	Two	Malting
53	Central	Abruzzo	Chieti	Furci	Planet	Two	Malting
56	Central	Umbria	Perugia	Montefalco	Sunshine	Two	Malting
57	Central	Umbria	Perugia	Marsciano	Sunshine	Two	Malting
58	Central	Umbria	Perugia	Papiano	Quench	Two	Malting
59	Central	Umbria	Perugia	Papiano	Ketos	Six	Feed
60	Central	Umbria	Perugia	Papiano	Alimini	Six	Feed

Table S10. *Fusarium* strains used to obtain standard curves for real-time quantitative PCR (qPCR) assays.

Species	Strain	References
Fusarium graminearum	FG8	127
Fusarium avenaceum	82dw09	127
Fusarium poae	56sw10	128
Fusarium culmorum	74dw09	127
Fusarium sporotrichioides	194dw13	111
Fusarium langsethiae	28PGBOSS	37
Fusarium proliferatum	149dw13	111
Fusarium tricinctum	27B14	22

Table S11. Primers used in quantitative real-time polymerase chain reaction (qPCR) assays.

Target	Primer name	Sequences (5’–3’)	References
Fusarium graminearum	Fg16N F	ACAGATGACAAGATTCAGGCACA	129
	Fg16N R	TTCTTTGACATCTGTTCAACCCA	
Fusarium avenaceum	Fave574	TATGTTGTCACTGTCTCACACCACC	130
	Fave627	AGAGGGATGTTAGCATGATGAAG	
Fusarium poae	FpoaeA51	ACCGAATCTCAACTCCGCTTT	130
	FpoaeA98	GTCTGTCAAGCATGTTAGCACAAGT	
Fusarium culmorum	FculC561	CACCGTCATTGGTATGTTGTGACTGG	130
	FculC614	CGGGAGCGTCTGATAGTCG	
Fusarium sporotrichioides	FspoS18	GCAAGTCGACCACTGTGAGTACA	130
	FspoA85	CTGTCAAAGCATGTCAGTAAAAATGAT	
Fusarium langsethiae	FlangA29	CAAGTCGACCACTGTGAGTACCTCT	130
	FlangA95	TGTCAAAGCATGTCAGTAAAGATGAC	
Fusarium proliferatum	Fpro220	CTTCGATCGCGCGTCCT	130
	Fpro270	CACGTTTCGAATCGCAAGTG	
Fusarium tricinctum	Ftri573	TTGGTATGTTGTCACTGTCTCACACTAT	130
	Ftri630	TGACAGAGATGTTAGCATGATGCA	
Translation elongation factor 1α (barley)	Hor1 F	TCTCTGGGTTTGAGGGTGAC	130
	Hor2 R	GGCCCTTGTACCAGTCAAGGT	
